# Genetic Mapping of Quantitative Trait Loci for Grain Yield under Drought in Rice under Controlled Greenhouse Conditions

**DOI:** 10.3389/fchem.2017.00129

**Published:** 2018-01-08

**Authors:** Julio Solis, Andres Gutierrez, Venkata Mangu, Eduardo Sanchez, Renesh Bedre, Steve Linscombe, Niranjan Baisakh

**Affiliations:** ^1^School of Plant, Environmental and Soil Sciences, Louisiana State University Agricultural Center, Baton Rouge, LA, United States; ^2^Instituto de Biotecnología, Universidad Nacional Agraria La Molina, Lima, Peru; ^3^Department of Biochemistry, University of Pennsylvania, Philadelphia, PA, United States; ^4^Center for Biotechnology Investigation, Escuela Superior Politecnica del Litoral, Guayaquil, Ecuador; ^5^Texas A&M AgriLife Research Station, Weslaco, TX, United States; ^6^Rice Research Station, Louisiana State University Agricultural Center, Crowley, LA, United States

**Keywords:** controlled condition, drought stress, QTL, rice, yield

## Abstract

Drought stress is a constant threat to rice production worldwide. Most modern rice cultivars are sensitive to drought, and the effect is severe at the reproductive stage. Conventional breeding for drought resistant (DR) rice varieties is slow and limited due to the quantitative nature of the DR traits. Identification of genes (QTLs)/markers associated with DR traits is a prerequisite for marker-assisted breeding. Grain yield is the most important trait and to this end drought yield QTLs have been identified under field conditions. The present study reports identification of drought yield QTLs under controlled conditions without confounding effects of other factors prevalent under natural conditions. A linkage map covering 1,781.5 cM with an average resolution of 9.76 cM was constructed using an F_2_ population from a cross between two Japonica cultivars, Cocodrie (drought sensitive) and Vandana (drought tolerant) with 213 markers distributed over 12 rice chromosomes. A subset of 59 markers (22 genic SSRs and 37 SNPs) derived from the transcriptome of the parents were also placed in the map. Single marker analysis using 187 F_2 : 3_ progeny identified 6 markers distributed on chromosomes 1, 5, and 8 to be associated with grain yield under drought (GYD). Composite interval mapping identified six genomic regions/quantitative trait loci (QTL) on chromosome 1, 5, 8, and 9 to be associated with GYD. QTLs located on chromosome 1 (qGYD1.2, qGYD1.3), chromosome 5 (qGYD5.1) and chromosome 8 (qGYD8.1) were contributed by Vandana alleles, whereas the QTLs, qGYD1.1 and qQYD9.1 were contributed by Cocodrie alelles. The additive positive phenotypic variance explained by the QTLs ranged from 30.0 to 34.0%. Candidate genes annotation within QTLs suggested the role of transcription factors and genes involved in osmotic potential regulation through catalytic/metabolic pathways in drought tolerance mechanism contributing to yield.

## Introduction

Rice is arguably the most important staple food that feeds more than half of the world population (Dhakarey et al., 2017). Modern semi dwarf rice varieties, a result of the green revolution, were selected primarily for high yield and resistance to biotic stresses, especially for irrigated ecosystems where available water is considered abundant for irrigation (Sandhu and Kumar, 2017). These varieties lacked resistance to abiotic stresses, such as water stress (drought, flooding, and submergence), problematic soil (salinity, alkalinity etc.), temperature extremes (heat, chilling, and freezing) and nutrient stress (deficiency and/or toxicity).

Rice is a profligate user of water, and it alone receives about 35% of the global surface water irrigation (Bouman et al., 2007). Erratic rainfall patterns due to the current and imminent environmental instabilities will increase the scarcity of water in arid and semi-arid regions and also are a great threat to the quality of water, where available, for crop use. Drought situation around the globe is apparent. In traditionally irrigated areas, such as in the U.S., drought is becoming an increasing concern, not only due to the climate change but also because of the demand for water for competing uses, which can lead to ground water paucity. In U.S. specifically, despite the present flooding situation in Houston 2017 and Louisiana in August 2016, overwhelming drought situations in the Southern U.S. (2010–2013), mid-western U.S. (2012–2013), in California (2014) are testimony to the fact that climate is changing and drought is inexorable. In addition, recurrent hurricanes in the coastal U.S. lead to the inundation of sea water into the cultivated area, thus making the irrigation water saline. The immediate effect of salinity is osmotic stress that makes the crops experience a physiological drought even with abundance of water available for irrigation. Thus, there is a need to develop rice varieties with drought resistance and acceptable yield.

Drought resistance is a complex phenomenon, which is the manifestation of both drought tolerance (tissue tolerance, maintenance of photosystem, etc.) and drought avoidance (deep root, leaf rolling, etc.) traits that are governed by multiple genes. Although several secondary drought resistance traits, such as green leaf area, canopy temperature, relative water content, etc. (Atlin and Lafitte, 2002; Richards et al., 2010) were identified to improve selection efficiency for yield under drought, complexity of the methods to phenotype the secondary traits limited their inclusion as a selection criterion in breeding. Also, expression of most loci governing these traits is influenced by both environmental conditions and genetic background, thus making difficult the assessment of their contribution to grain yield. Therefore, direct selection for yield was considered the most successful approach to improve drought resistance in rice (Kumar et al., 2008). Major criteria to evaluate the performance of genotypes against drought are drought score, grain yield and spikelet fertility (Swain et al., 2014). However, data accumulated in the past suggest that drought tolerance for yield components is largely associated with genetic and physiological factors independent from those determining the traits *per se* (Lang et al., 2013).

Genetic mapping of quantitative trait loci (QTLs) to identify genomic regions controlling grain yield under drought and subsequent utilization of markers linked to the QTLs in marker-assisted pyramiding of desirable alleles is an effective alternative to breeding high yielding drought resistant rice (Ashraf, 2010). Despite limited success of using QTLs in molecular breeding due to the repeatability of QTLs (Bernier et al., 2008), significant progress has been made toward mapping QTLs for drought resistance traits, such as morphological, physiological and yield related traits in rice (Swamy et al., 2017) by the integration of molecular markers and sequencing technologies in rice breeding studies (Thomson et al., 2010; Swamy et al., 2014).

Most of the yield related QTLs reported in the literature came from studies that focused on different components associated with yield rather that grain yield under drought stress from studies involving drought resistant local landraces and ecotypes (Ikeda et al., 2013; Sun et al., 2013; Yan and Bao, 2014). The QTL, qDTY12.1, located on chromosome 12, was the first QTL reported for rice grain yield in upland reproductive-stage drought stress conditions (Bernier et al., 2007). It's contribution to improve grain yield under drought tolerance is significant in terms of the genetic variance explained (43 to 51%) and its consistent effects in multiple environments (Bernier et al., 2007, 2009a,b; Boopathi et al., 2013). qDTY12.1 is the only locus utilized to generate drought resistant lines with increased grain yield for highly diverse upland and lowland rice ecosystems (Bernier et al., 2007, 2009a; Mishra et al., 2013); however, map-based cloning of qDTY12.1 to identify causal gene(s) is not yet available. QTL, qDTY1.1 on chromosome 1, with 8.55–30% additive effects, is one of the most recent examples of major QTLs discovered for grain yield under drought identified in different genetic backgrounds and from different donors, with the positive allele coming from the drought resistant varieties N22 (Vikram et al., 2011) and Dhagaddeshi (Ghimire et al., 2012). Two other QTLs, qDTY2.3 on chromosome 2 and qDTY3.2 on chromosome 3 (Tang et al., 2013) derived from Vandana cultivar, interact with qDTY12.1 to enhance yield and harvest index of qDTY12.1-positive lines under severe upland and lowland drought conditions (Dixit et al., 2012). qDTY2.3 had a large effect, accounting for 18–31 % (*R*^2^) improved grain yield under severe lowland stress (Yadaw et al., 2013; Saikumar et al., 2014). qDTY3.2 also accounted for variation in canopy temperature during flowering and seedling shoot dry weight under stress and drought recovery (Saikumar et al., 2014). Likewise, Zhang et al. (2014) reported five stably expressed QTLs (*QSnp1b, QGyp2a, QSnp3a, QSf8, and QSnp11)* from a set of 20-30 QTLs for drought resistance-related traits in a cross between Lemont (drought sensitive) and Teqing (moderately drought tolerant). QTL *QSnp1b* and *QGyp2a* mapped with qDTY1.1 (Vikram et al., 2011) and qDTY2.1(Venuprasad et al., 2009), respectively, whereas other QTLs localized in chromosomal regions harboring genes for abiotic stress response and hormone signaling (Ye et al., 2009; Jeong et al., 2013). Five QTLs on chromosomes 1, 6, 8, 10, and 12 with 2.47–13.89% effect on grain yield under drought were identified (Lang et al., 2013) with the largest effect QTL on chromosome 12 coincided with a minor QTL for grain thickness (Zhang et al., 2013). Sixteen drought grain yield QTLs distributed over all chromosomes except 5, 7 and 8 were reported in rice at IRRI (Kumar et al., 2014). Meta-QTL analyses of 15 populations identified 14 MQTLs on seven chromosomes (four on chromosome 1, two each on chromosomes 2, 3, 8, and 10, and one each on chromosomes 4 and 12), with 4–28% effect on drought grain yield (Swamy et al., 2011). This study showed that the qDTY12.1 was present in 85% of the lines, whereas qDTY1.1, qDTY1.2, qDTY3.2, qDTY4.2, and qDTY8.1 were present in >50% of the lines.

In the southern U.S., rice breeding programs have been focused to improve grain yield and quality with resistance against pathogen and insect pests. Except for a few reports (De Leon et al., 2015, 2016), limited attention has been paid to understanding the genetics toward breeding for abiotic stress tolerance. Evaluation of the impact of drought on grain yield under field conditions is an ideal situation to dissect genes/QTLs associated with drought tolerance, but it requires a dry season, a factor that is beyond researcher's control. Therefore, the present study was undertaken under controlled greenhouse. This is the first study, to our knowledge, that reports identification of genetic factors controlling grain yield under drought using the southern U.S. cultivar with an objective to identify molecular markers associated with QTLs for rice grain yield under drought conditions.

## Materials and methods

### Mapping population

The mapping population comprised of 187 F_2 : 3_ families developed from individual F_2_ plants derived from selfing of the F_1_s of a cross between “Cocodrie” (female recipient) and “Vandana” (male donor). “Cocodrie” (PI 606331) is a high-yielding (8.5 t/ha), semi-dwarf, early maturing and long grain rice variety, originated from the cross “Cypress”//”L-202”/ “Tebonnet” (Linscombe et al., 2000). It is sensitive to drought stress and moderately resistant/susceptible to different blast races. “Vandana,” derived from the cross “C-22”/”Kalakeri” cross, is a short duration (95 days) tall cultivar with a yield potential of 3.5 t/ha. Because of its drought tolerance, in addition to moderate resistance to sheath blight and blast, it was released by the National Rice Research Institute, India in 1992 for cultivation in rainfed upland ecosystem of eastern India (http://drdpat.bih.nic.in).

### Drought phenotyping

One hundred eighty seven F_2 : 3_ families and the parents were grown in 4 L plastic pots filled with soil:potting mix (2:1) inside the greenhouse that was maintained at 29°C/21°C day/night temperature regime and 60–70% relative humidity maintained through a cool misting pad on a side wall of the greenhouse compartment. Twelve plants per family with two plants in each pot were grown. The pots were placed on ceramic trays covered with plastic films. Irrigation was applied with tap water to the tray at 6 cm twice in a week to maintain the pots at 100% field capacity (soil moisture content 0.48 m^3^/m^3^).

Three pots (six plants) from each family and parents with 50 days-old plants were transferred to three plastic-covered ceramic trays (three blocks) and kept for 15 days without watering to impose drought stress. The soil moisture content after 7 days was 0.11 to 0.14 m^3^/m^3^ and after 14 days was 0.06 to 0.08 m^3^/m^3^. Following drought, irrigation was resumed until physiological maturity of the grains. The other three pots (six plants) per family received regular watering in the trays (control). Weight of fully filled grains (grain yield) was taken for individual plants and averaged per family. The experiment was conducted in fall 2011 and spring 2012.

### Statistical analysis

Frequency distribution of the grain yield under drought was charted as histogram using basic R v3.4.1 package. Analysis of variance (ANOVA) of the yield data was estimated as described earlier (Gutierrez, 2016) using mixed model (Proc MIXED) in SAS 9.3 (SAS Institute, 2011). Broad-sense heritability was calculated on family means basis (ceramic trays as blocks) using ANOVA-derived variance components as per Hallauer and Miranda (1981).

### Molecular markers

Three hundred thirty simple sequence repeat (SSR) markers (McCouch et al., 2002) and 33 Indel markers (Wu et al., 2013) were screened for polymorphism between the parents, Cocodrie and Vandana. In addition, 37 genic SSRs from the differentially expressed unigenes of the transcriptome of Cocodrie and Vandana (Supplementary Table S1) were used. Primer pairs were designed using Primer3 (http://primer3.ut.ee) for unigenes containing SSRs with at least 5 of di-nucleotide, 4 of tri nucleotide, 3 of tetra-nucleotide or 2 of penta-nucleotide repeat motifs. SSRs were designed from selected sequences that did not match to SSR-containing sequences from the Gramene database (www.gramene.org). Also, 120 SNPs spanning 12 chromosomes were selected randomly from Cocodrie and Vandana transcriptomes for parental polymorphism screening (Supplementary Table S2) with the allele-specific primers designed for each SNP using SNAPER tool (Drenkard et al., 2000). Location of all markers were assigned in reference to the pseudomolecules of MSU Rice Genome Annotation Project Release 7 (Ouyang et al., 2007).

### Genotyping and marker analyses

DNA was extracted from freshly harvested leaves of 187 individual F_2_ individuals using the CTAB method (Khan et al., 2013). Fifty nanogram DNA was subjected to polymerase chain reaction in a total volume of 10 μl that contained 1.0 μl 10x PCR buffer, 1.0 μl 25 mM MgCl2, 0.2 mM dNTP, 25 ng each of forward and reverse primers, and 0.4 U Taq polymerase (Promega, Madison, WI). Thermal profile for SSRs was: initial denaturing at 94°C for 4 min, followed by 35 cycles of 94°C for 30 s, 55°C for 45 s, and 72°C for 1 min, with a final extension at 72°C for 5 min. SNP-based genotyping was performed using SNAP method as described by Drenkard et al. (2000). The PCR conditions for the SNPs were: polymerase activation at 94°C for 5 min, followed by 35 cycles of 94°C for 30 s (ramping of 2°C/s), 66°C for 1 min (ramping of 1.4°C/s). The amplification products of SSR and SNP markers were resolved in 4.5% SFR and 2% biotechnology-grade agarose (Amresco, Solon, OH) gels, respectively.

### Linkage and QTL mapping

Multipoint linkage analysis was performed with 187 F_2_ lines with 213 markers (171 SSRs, 5 InDels, and 37 SNPs) that were evenly distributed over 12 rice chromosome. The map position of each marker in centimorgan (cM) was estimated from the observed recombination frequencies (r) using the Kosambi mapping function as implemented by the software MAPMAKER3.0 (Lander et al., 1987). The framework map order was determined with a logarithm of odd (LOD) of 3.0 set as the threshold for significance. Further, preferred order of markers with different positions on entire chromosome was checked by the “ripple” command, and the position of markers were assigned taking into account the expected location based on the rice physical map (IRGSP, 2005).

QTL analysis with the average yield data of the F_2 : 3_ families was carried out by single marker regression analysis using R/QTL (Arends et al., 2010) at a LOD threshold of 2.5. Interval mapping (IM) and composite interval mapping (CIM) were conducted using WinQTL Cartographer version 2.5 (Wang et al., 2012) with 1,000 permutations. CIM was performed using genome wide error (α) = 0.05 and LOD threshold of 2.2. QTL analysis was also performed with MAPQTL5 (Van Ooijen, 2004) and QTL ICIM (Wang et al., 2016). QTLs that were consistent across all software platforms were taken into consideration and called significant if the phenotypic variance explained (PVE) was at least 5% and/or the LOD was ≥2.2. Standard nomenclature, e.g., “q” followed by the abbreviate phenotype “GYD” (grain yield under drought) and chromosome number (1, 2, 3….12) and the QTL number (e.g., qGYD1.1, qGYD1.2…) was given for a QTL. The peak, map position, confidence interval, PVE, additive (A) and dominance (D) components for each QTL were retrieved from the analysis. Positive and negative values of the QTLs corresponded to phenotypic variance contributed by Vandana and Cocodrie, respectively. The D/A ratio of 0–0.20, 0.21–0.80, 0.81–1.20, or >1.20 explained for Additive (A), partial dominance (PD), dominance (D) and overdominance (OD) mode of gene action as described by Stuber et al. (1987).

### Identification of candidate genes in QTL region

Chromosome positions of the markers flanking the QTLs were used to identify the genes within QTL intervals using SNP-Seek II (Mansueto et al., 2017). Genes were compared to the set of genes that were significantly differentially expressed from the transcriptome study of Cocodrie vs. Vandana, and only common genes were considered for gene ontology analysis using GOSlim (Bedre et al., 2016).

## Results

One hundred seventy three out of 330 SSRs selected from the Gramene database showed polymorphism between the parents, Cocodrie and Vandana. However, 24 SSRs produced complex banding pattern, similar-sized alleles or skewed segregation among the F_2_ individuals, and thus were excluded from further analysis. Only 5 out of 33 Indels generated distinct polymorphic bands between the parents. Of the 37 genic SSRs, 22 SSRs were confirmed being polymorphic with good resolution between the parents. Similarly, 37 out of 120 SNPs were polymorphic without cross-amplification between the parents. Thus, a total of 213 markers showing distinct, reproducible polymorphism between the parents were used for mapping.

### Grain yield variation under controlled greenhouse conditions

**Figure 1:**
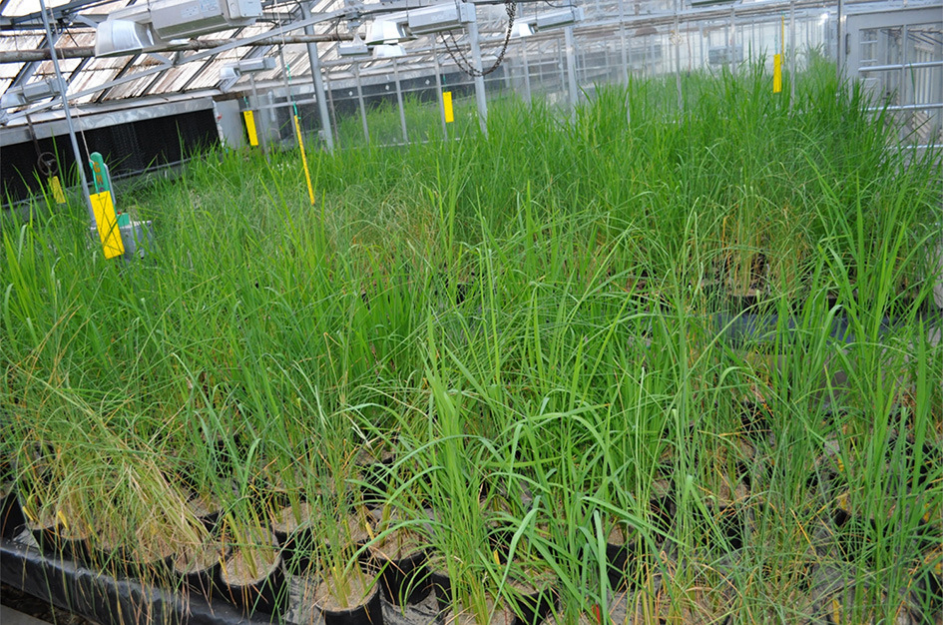
F_2 : 3_ progenies of Cocodrie × Vandana showing differential phenotypic symptoms under drought stress in controlled greenhouse conditions.

**Figure 2:**
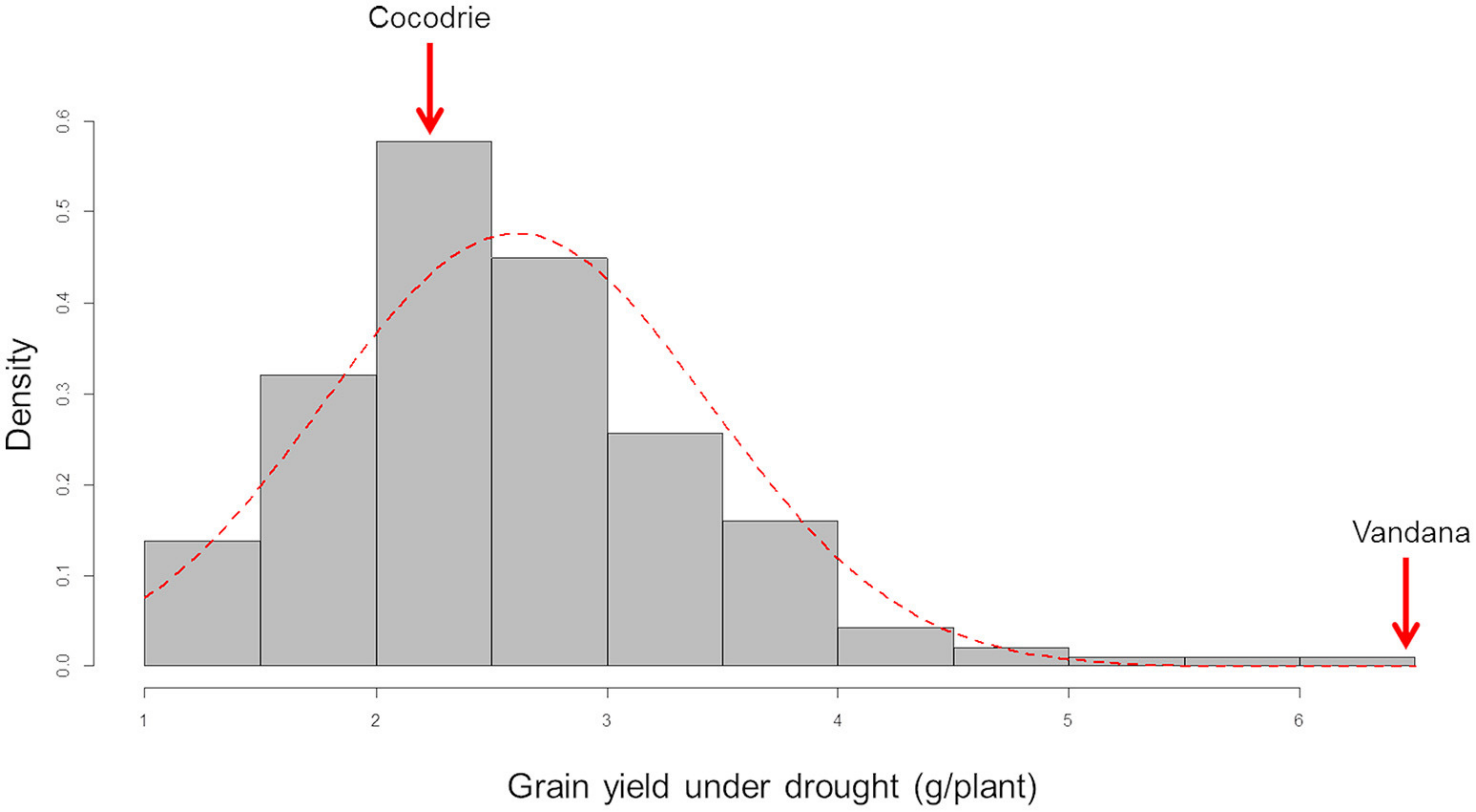
Frequency distribution of the grain yield of F_2 : 3_ progenies of Cocodrie x Vandana under greenhouse drought conditions. Density frequency/interval.

The parents and the F_2 : 3_ lines showed differential response with typical symptoms, such as leaf wilting, rolling and drying under drought stress after a week of withholding water (Figure 1). Average grain yield per plant of the drought stressed F_2 : 3_ lines ranged from 1.08 (±0.31) to 6.27 (2.02 SD) g. The parents, Cocodrie and Vandana yielded 2.23 (±1.01) g and 6.70 (±1.99) g per plant, respectively. The yield per plant of the parents under well-watered conditions was 7.59 (1.08) g and 14.83 (3.04) g, respectively, while the yield of the F_2 : 3_ lines ranged from 2.03 (0.09) g to 12.99 (3.11) g. The phenotypic distribution of the grain yield under drought followed a near-normal distribution (Figure 2). The broad sense heritability of the grain yield under drought was moderately high (0.28) considering that yield is a quantitative trait (Supplementary Data S1).

### Linkage map and QTL analysis

The linkage map developed using 213 markers including the new 59 markers (22 genic SSRs + 37 SNPs; Supplementary Tables S1, S2) was 1,781.5 cM long with an average distance of 9.76 cM between the adjacent markers (Supplementary Figure S1). Chromosome 1 (216.8 cM) was the longest while chromosome 10 (54.3 cM) was the smallest. The markers were evenly distributed across all 12 chromosomes with some gaps, especially in some regions of chromosome 1, 2, 4, 6, 7 and 10. For example, chromosome 4 located between RM16649 (13.5 Mb) and RM3643 (20.1 Mb), chromosome 6 had a gap between markers RM20048 (16.1 Mb) and RM5371 (25.8 Mb) and chromosome 7 had the largest gap between markers RM1353 (3.31 Mb) and RM3449 (13.4 Mb).

#### (Composite) interval mapping

**Figure 3:**
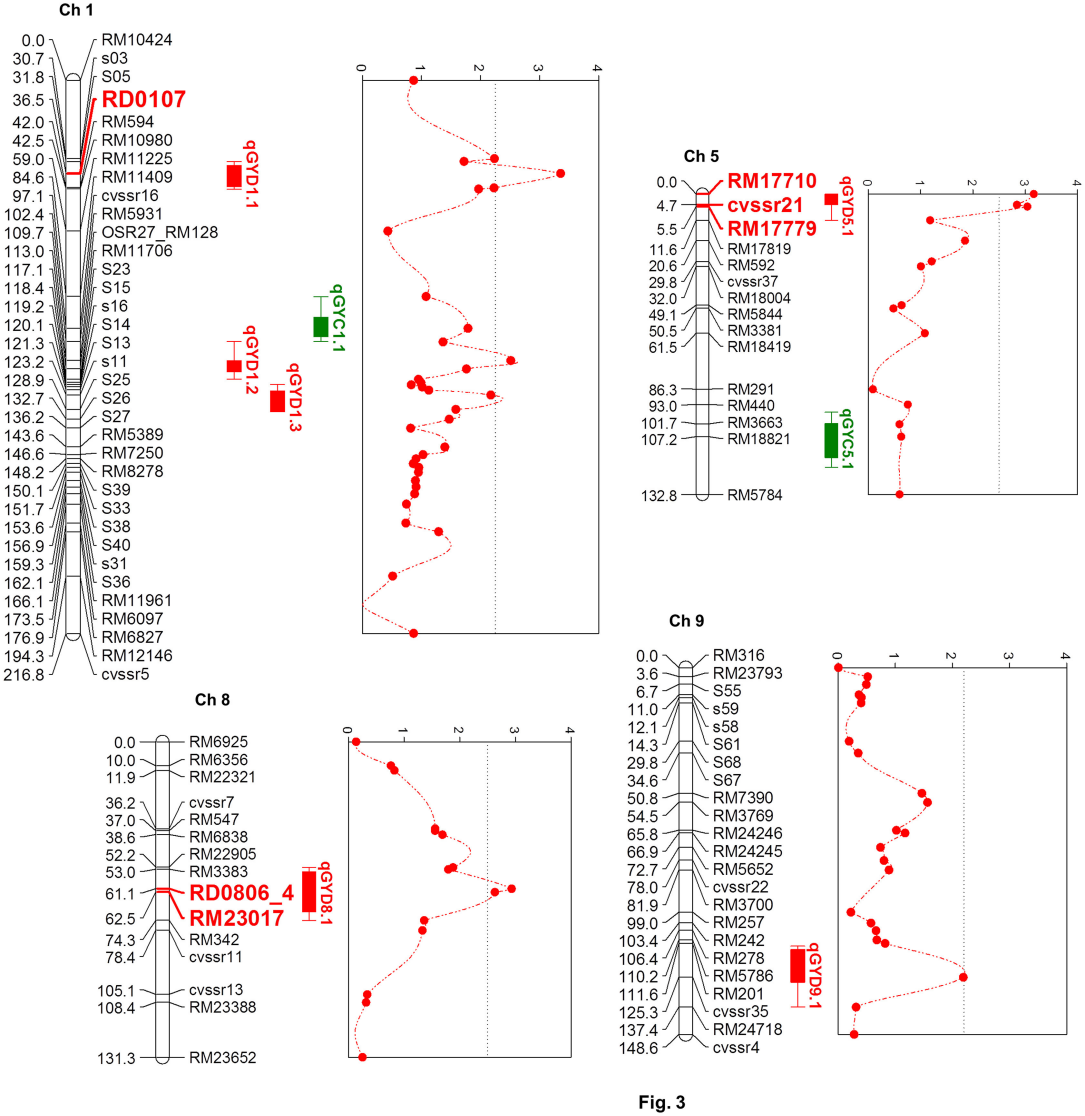
Quantitiative trait loci on chromosomes 1, 5, 8, and 9 associated with grain yield under greenhouse drought. QTLs (in green) represent the genomic regions associated with grain yield in non-stressed control conditions. Markers identified through single marker analysis and within the QTL interval are depicted in bold red fonts.

Six chromosomal regions were identified from the genome-wide scan of the F_2 : 3_ families, which harbored the QTLs controlling grain yield under drought (Table 1; Figure 3). Three QTLs were on chromosome 1 (qGYD1.1, qGYD1.2, qGYD1.3) and one each was on chromosome 5 (qGYD5.1), 8 (qGYD8.1), and 9 (qGYD9.1). The phenotypic variance explained (PVE) by the QTLs ranged from 5.00% (qGYD1.3) to 7.23% (qGYD5.1). QTLs, qGYD1.1 (PVE = 7.10%) and qGYD9.1 (6.10%) had overdominance effects of the alleles contributed by Cocodrie parent. QTLs, qGYD1.2 and qGYD1.3 were close to each other (14.1 bin) that had increasing additive effect contributed by alleles from Vandana. Similarly, QTLs on chromosome 5 (qGYD5.1) and 8 (qGYD8.1) had additive effects (33% and 34%, respectively) on grain yield under drought with total PVE 7.23 and 6.14%, respectively that came from Vandana alleles. qGYD5.1 had the peak on the proximal tip of the chromosome (Figure 3).

**Table 1:** QTL analysis of grain yield under drought stress in F_2 : 3_ progenies of Cocodrie × Vandana.

**QTL**	**Chr.**	**Peak position (cM)**	**Marker interval**	**Bin**	**LOD**	**PVE (%)**	**Add. effect**	**Dom. effect**	**Gene action**	**Confidence interval**
qGYD1.1^a^	1	36.5	RD0107–RM594	5.5	3.6	7.10	−0.04	−0.46	OD	33.25–41.55
qGYD1.2^a^	1	110.7	RM128–RM11706	3.3	2.6	7.00	0.32	−0.26	Add.	109.75–114.15
qGYD1.3^b^	1	124.8	S11–S25	5.7	2.4	5.00	0.30	−0.32	Add.	121.65–129.55
qGTD5.1^a^	5	0	RM17710–cvssr21	4.7	3.2	7.23	0.33	−0.03	Add.	0.00–5.50
qGTD8.1^a^	8	61.1	RD0806_4–RM23017	1.4	2.8	6.14	0.34	−0.07	Add.	54.05–70.65
qGYD9.1^b^	9	123.7	RM201-cvssr35	13.7	2.2	6.10	−0.18	−0.28	OD	114.05–132.05

*Chr., chromosome; cM, centi Morgan; Mbp, mega base pair; LOD, logarithm of odds; PVE, phenotypic variance explained; add., additive; dom., dominance; OD, overdominance*.

a *QTL identified in both 2011 and 2012*.

b *QTL identified in only 2012*.

The markers identified by SMA corresponded to the QTLs identified by composite interval mapping. Except for RM17779, five markers identified in chromosomes 1, 5, 8, and 9 from SMA were linked to the QTLs by CIM. The two markers (RM17710 and cvssr21) on chromosome 5 identified by SMA delimited the QTL, qGYD5.1. The only exception was that no marker was identified by SMA on chromosome 9, whereas qGYD9.1 at a low LOD (2.2) was identified with an overdominance effect on the grain yield under stress. Expectedly, the gene action of the QTLs by CIM and markers by SMA (Supplementary Table S3) had the same mode of gene action, further validating the mutual agreement of the analysis.

**Figure 4:**
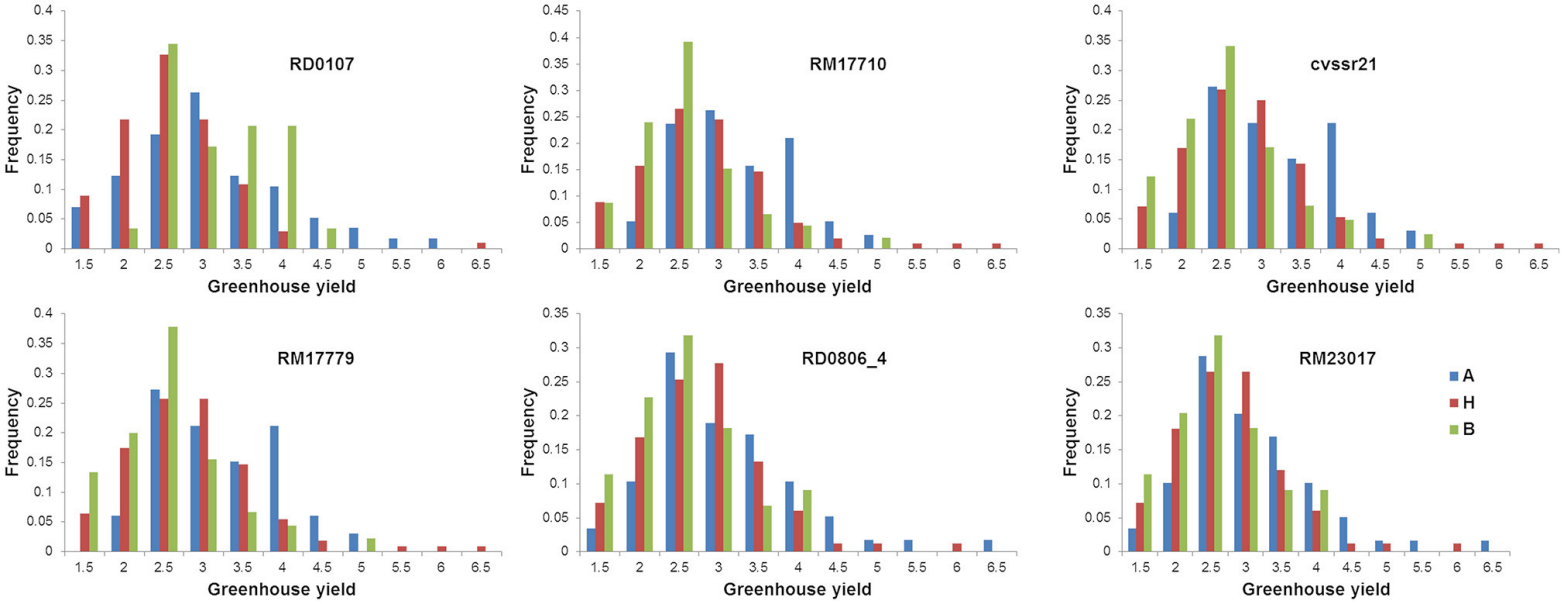
Genotypic frequency of the F_2 : 3_ progenies of Cocodrie × Vandana at locus associated with grain yield under greenhouse drought. A, Cocodrie; H, heterozygous; B, Vandana.

Genotype frequency for the average yield under drought of the F_2 : 3_ lines were calculated at the markers closest to the QTLs. The mean yield of the lines homozygous for the Cocodrie allele at the marker (RD0107) closest to qGYD1.1 was higher than the lines homozygous for the Vandana allele (Figure 4). On the other hand, the mean performance of the lines for the Vandana allele was relatively higher at other markers closest to qGYD1.2, qGYD1.3, qGYD5.1, and qGYD9.1.

### Differentially expressing gene candidates within QTL region

**Figure 5:**
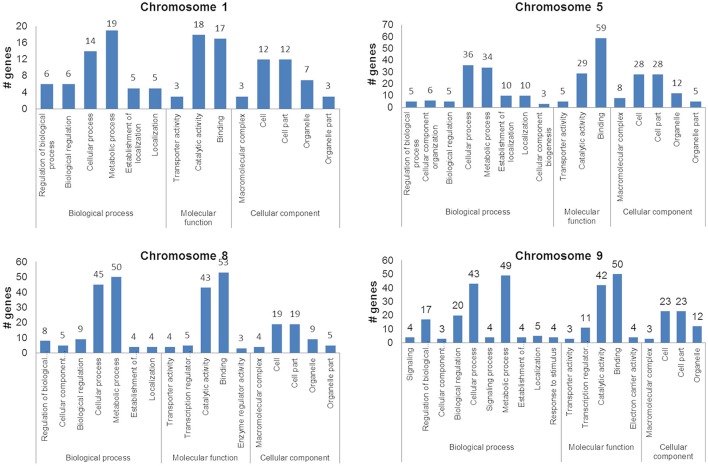
Ontology of the candidate genes within the quantitative trait loci associated with grain yield of the F_2 : 3_ progenies of Cocodrie × Vandana under greenhouse drought conditions.

Genes within the QTLs were identified by taking into account the physical location of the QTL interval markers. Comparison of the genes against differentially expressing genes from the comparative transcriptome of the Cocodrie and Vandana reduced the number of candidate causative genes underlying the expression of QTLs. The total number of genes identified within the QTL regions, after excluding the expressed/hypothetical proteins and transposons/retrotransposons were 289 (chromosome 1–38, chromosome 5–74, chromosome 8–115, and chromosome 9–62) (Supplementary Data S2). GO enrichment analysis showed that most of the genes were involved in metabolic and cellular processes with DNA binding and catalytic activity in the cytosol or organelles. Transcription factors and genes coding for hydrolase/transferase were abundant in cellular and metabolic processes primarily in the cell (Figure 5).

In general, Cocodrie and Vandana shared 532 significantly up-regulated and 267 significantly down-regulated genes (log2FC≥2; *P* < 0.05). On the other hand, Vandana had 273 (up) and 364 (down) unique genes as compared to 844 (up) and 532 (down) in Cocodrie. Most of these genes were known to be implicated in general stress response, specifically regulated under drought stress. Of significance were the genes involved in ABA biosynthesis and pathway (9-cis-epoxycarotenoid dioxygenase, aldehyde oxidase and dehydrogenase/reductase), antioxidant biosynthesis (superoxide dismutase, peroxidase, catalase and glutathione-S-transferase), osmoprotection (beta-amylase, trehalose phosphate synthase, glutamate decarboxylase), and transcription factors (DREB1A, ethylene response factor, NAC1, bHLH, CCCH-zinc finger protein), kinases/phosphatases (CDPK, CBL, CIPK, protein phosphatase 2C, sucrose non-fermenting 1-related protein kinase). In addition, other known stress resistance genes, such as those coding for late embryogenesis abundance proteins, heat shock proteins, dehydrin, early-responsive to dehydration protein were significantly differentially expressed under drought in Cocodrie and Vandana.

## Discussion

The present study targeted grain yield as it is the main focus of current rice breeding efforts in the development of improved drought resistant rice varieties (Venuprasad et al., 2012a,b; Dixit et al., 2014; Saikumar et al., 2014). By using grain yield as the selection criterion, we detected six QTLs that primarily control mechanisms toward higher yield under drought using F_2 : 3_ lines of two parental cultivars, Cocodrie and Vandana with contrasting drought response. Involvement of other genetic mechanisms, such as those underlying drought avoidance and plant recovery following stress, contributing to the yield cannot be overruled as leaf rolling was observed among the progeny. Grain yield (weight) is the result of the sum of carbohydrate accumulated before heading and translocated/loaded after heading (Swain et al., 2014), and thus is primarily determined by highly correlated traits, such as panicle number per plant, spikelet number per panicle, and spikelet fertility. It is not surprising that loci associated with these traits have been reported co-localized under both irrigated and drought stressed conditions (Venuprasad et al., 2012b; Mishra et al., 2013; Saikumar et al., 2014). Therefore, grain yield was the only trait that was measured under drought in the present study.

QTLs for grain yield under drought in managed stress environments, such as controlled greenhouse conditions may not be translatable to target field environments where the timing and severity of drought vary over years (Prince et al., 2015), and such conditions will change the responses of plant's traits involved in drought-resistance mechanisms (Kamoshita et al., 2008). However, under circumstances where a drought experiment under field conditions with unpredictable rainfall pattern and lack of a rainout shelter is a distant reality, simulated drought under greenhouse studies could provide important primary information on the heritability of the trait and chromosomal regions associated with grain yield by near-exact manipulation of the water regime. In addition, greenhouse conditions will eliminate the edaphic variability (soil texture, soil temperature, etc.) and other environmental confounding factors (humidity, biotic stresses) that hamper precise phenotyping under natural field conditions and detection of causative QTLs. In the present study, the broad sense heritability was moderate (28%), which is similar to the results obtained under controlled conditions by Manickavelu et al. (2006).

Using 59, not previously reported markers (22 SSRs and 37 SNPs), along with 159 SSRs and five indels for genetic mapping, we identified six QTLs for grain yield in chromosome 1, 5, 8, and 9 under drought, whereas no QTL for drought grain yield was found on chromosomes 5 and 8 out of the 16 QTLs reported by Kumar et al. (2014) and 7 meta-QTLs identified by Swamy et al. (2011). Markers RD0107, RM594, and OSR27_RM128 associated with grain yield under drought QTLs, qGYD1.1 and 1GYD1.2, in our study were located at 12.9 Mb, 15.15 Mb and 30.7 Mb of chromosome 1, respectively and were far from qDTY1.1 (Vikram et al., 2011). However, qGYD1.1 and qGYD1.2 co-localized with minor effect QTLs for drought tolerance and drought avoidance traits, such as measuring fitness, yield, and the root system identified from experiments carried out in PVC pipes-grown plants with Zheshan 97/IRAT109 progeny (Yue et al., 2006). Marker RM 128 flanking qGYD1.2 in our study co-located with metaQTL, mqMRL_1-2 at 32.5 Mb for maximum root length, and other QTLs for root thickness, penetrated root thickness and penetrated root length (Courtois et al., 2009). Similarly, marker RM128 was also found to flank a QTL, *ph1.4* for plant height with RM226 (Kaladhar et al., 2008), which suggested pleiotropic effect of a chromosomal region affecting grain yield and plant anatomical features. The effect of qGYD1.1 (Table 1) appeared to be dependent on genetic background, as the effect was derived from the drought sensitive Cocodrie cultivar.

Although a region on chromosome 5 between SNP markers S4134205–S7643153 was found harboring four QTLs for grain yield and related traits in two genetic backgrounds and different environments (Wang et al., 2014), this segment did not overlap with the QTL, qGYD5.1 (RM17710-cvssr21) identified in our study. On the other hand, a minor, inconsistent QTL for 100 grain weight under drought was reported on chromosome 5 (RM509-RM430) in the Zhenshan 97/IRAT109 population (Yue et al., 2006).

A QTL on chromosome 8 (qtl 8.1) with the marker interval RM337-RM3664 and peak at RM8020 was found to be associated with grain yield under well-watered upland conditions (Bernier et al., 2007) that overlapped with metaQTL MQTL8.1 (RM337-RM902) for drought grain yield (Swamy et al., 2011) around RM25 reported in Apo/IR72 and Vandana/IR64ApoIR (Venuprasad et al., 2012a). QTLs associated with yield, spikelet fertility and root traits of drought plants relative to control plants were identified in chromosome 8 using Zhenshan 97/IRAT109 (Yue et al., 2006). The QTL, qGY8.1 for grain yield under aerobic conditions (Sandhu et al., 2013) and qDTY8.1 under drought stress (Vikram et al., 2012) co-localized between RM339 and RM210, close to regions harboring QTLs for other yield related traits and root length (Sandhu et al., 2013). Genome-wide association studies also identified the marker RM6070 of chromosome 8 to be significantly associated (*P* < 0.01) with both plant height (*R*^2^ = 3.96) and percentage seed set (*R*^2^ = 12.85%) (Zhou et al., 2012). The markers, RD0806_4 and RM23017, linked to qGYD8.1 identified to be associated with grain yield under drought (*R*^2^ = 6.4) from our study overlapped with the MQTL8.2 (Swamy et al., 2011). A major QTL located on chromosome 8 explaining 54% of the phenotypic variance for grain yield was reported in Swarna × *O. nivara* population (Kaladhar et al., 2008). All these reports indicate that chromosome 8 could be a hot spot for alleles with positive effect on yield and drought recovery traits.

The qtl9.2 for biomass yield involving Vandana/Way Rarem was delimited by RM201 and RM205 (Bernier et al., 2007), which overlapped with qGYD9.1 (RM201-cvssr35) for grain yield under drought from our study. However, the positive allele was contributed by Cocodrie in our study as opposed to Vandana that contributed the positive allele in the study by Bernier et al. (2007). Using the advanced backcross lines at seedling stage, a QTL for increased root length and drought tolerance was mapped in the segment between RM201 and RM328 of chromosome 9 (Lang et al., 2013), which was close to the Dro1-KP allele for deep rooting (Uga et al., 2013). In addition, qDTY9.1 for grain yield under drought was flanked by RM105-RM434 (Swamy et al., 2013), which is the single QTL on chromosome 9 for enhanced yield under lowland conditions (Kumar et al., 2014). Chromosome 9 has been documented to harbor hot spots for QTLs for maximum root length and other root traits detected in multiple environments and in different genetic backgrounds (Courtois et al., 2009). The qGYD9.1 detected in our study co-localized with meta-QTLs at ~20 Mb for root traits, such as maximum root length, root thickness and deep root weight (Courtois et al., 2009). Drought avoidance and drought tolerance mechanisms appear to be the underlying effects of the QTLs on chromosome 9 toward improved grain yield, thus emphasizing the necessity to pyramid these traits.

Other QTLs, previously reported in populations involving Vandana as a parent were qDTY6.1 on chromosome 6 (Venuprasad et al., 2012a) under aerobic drought stress and irrigated lowland conditions, and qtl2.1 on chromosome 6 with significant effects on grain yield under non-stress conditions (Bernier et al., 2007). However, no QTL was found on chromosome 6 associated with drought grain yield in the mapping population in our study. Interestingly, our greenhouse drought study did not identify a QTL on chromosome 12, unlike qtl12.1 that was identified in F_3_ generations of Vandana/Way Rarem (Bernier et al., 2007) with the positive allele for high yield under drought contributed by the drought sensitive Way Rarem. However, qGYC12.1, a QTL for grain yield under non-stressed control with ~50% additive effect (PVE 4.8%) was identified in our study (Supplementary Figure S1; Supplementary Data S3). QTLs identified for yield under unstressed control conditions did not overlap with any of the drought grain yield QTLs in our study. Since, the present work was conducted to dissect loci with effect on grain yield under controlled conditions, it is not surprising that QTLs reported here do not overlap/co-localize with the reported QTLs coming from studies carried out under upland and lowland drought situations. However, QTLs, such as DRO1 for drought tolerance or drought avoidance have been found in controlled conditions (Uga et al., 2011). In most studies involving phenotypic evaluations of drought tolerance under upland and lowland conditions across multiple environments, few grain yield QTLs have shown consistent effects. Drought tolerance QTLs detected for upland rice varieties may not be suitable for rainfed lowland conditions and vice versa (Mackill et al., 1996), mainly because of the unique hydrology of rainfed lowlands in which soil transitions from flooded and anaerobic to drought and aerobic (Wade et al., 1999).

Due to the complexity of the components involved in grain yield under drought, many QTLs for different yield-related traits co-localize in same chromosomal regions with pleoiotropic effects. Although, many genetic studies have revealed the location of QTLs of interest for grain yield, only a few reported the genes and their mode of action within the discovered QTLs (Ashikari et al., 2005). The QTLs/genes presented here are expected to provide further clues to identifying underlying mechanisms involved in improved grain yield under drought stress. Our on-going experiments are aimed at confirming the genomic regions and narrowing down of the number of genes reported within the QTLs in the present study through comprehensive studies involving high-resolution linkage mapping via high-throughput genotyping by sequencing of advanced generation progeny such as RILs/BILs and phenotyping under natural conditions with rain-out shelter facilities.

## Author contributions

NB: conceptualized and designed the experiment; NB, VM, JS, ES, and SL: performed the experiments; JS, AG, and RB: analyzed the data; JS and NB: wrote the manuscript.

### Conflict of interest statement

The authors declare that the research was conducted in the absence of any commercial or financial relationships that could be construed as a potential conflict of interest. The reviewer, MK, and handling Editor declared their shared affiliation.

## Supplementary material

Supplementary Table S1 Genic SSR markers derived from the Cocodrie and Vandana transcriptome used for mapping of Cocdrie/Vandana F2 progenies.

Supplementary Table S2 Single nucleotide polymorphism (SNP) markers placed in the genetic map of Cocodrie/Vandana F2 progenies.

Supplementary Table S3 Markers associated with grain yield under drought stress in F2:3 families of Cocodrie/Vandana from single marker analysis and mean grain yield (g) per plant on each genotype (A, homozygous for Vandana alleles; B, homozygous for Cocodrie alleles; H, heterozygous).

Supplementary Data S1 Analysis of the grain yield of F2:3 progenies of Cocodrie × Vandana under greenhouse drought conditions.

Supplementary Data S2 Genes within QTLs on chromosomes 1, 5, 8, and 9.

Supplementary Data S3 Quantitative trait loci identified for grain yield under non-stressed control using Cocodrie/Vandana F2:3 families.

Supplementary Figure S1 Linkage map of the F2 individuals showing distribution of the markers on 12 chromosomes. QTLs for grain yield under greenhouse drought are shown in red rectangles and QTLs for grain yield under non-stressed control are in green rectangles. Markers identified through single marker analysis and within the QTL interval are depicted in bold red fonts.
