# Supplementary material for: Genetic Mapping of Quantitative Trait Loci for Grain Yield under Drought in Rice under Controlled Greenhouse Conditions
Source: Front Chem. 2018 Jan 8;5:129. doi: 10.3389/fchem.2017.00129 (PMC5766644; doi:10.3389/fchem.2017.00129)
Supplement: Supplementary Table S1 — Genic SSR markers derived from the Cocodrie and Vandana transcriptome used for mapping of Cocdrie/Vandana F2 progenies. [file Table1.DOCX]

**Supplementary Table S1**. Genic SSR markers derived from the Cocodrie and Vandana transcriptome used for mapping of Cocdrie/Vandana F_2_ progenies.

| **Genic SSR** | **Forward primer**  **(5’ – 3’)** | **Reverse primer**  **(5’ – 3’)** | **Alleles in V/C (bp)** | **Motif (in Vandana)** | **Chr** | **In Map** | **Coordinate** | **Amplicon (bp)** |
| --- | --- | --- | --- | --- | --- | --- | --- | --- |
| cvssr1 | ctttgagggatcgtgagcaat | gcgacttcaacttctcctcc | 194/185 | (GCG)5 | Chr3 | yes | 2337029 | 185 |
| cvssr2 | catcaagtccttctccaccac | ctacgaaaataaacccccaca | 220/211 | (CCA)5-(CGA)5 | Chr3 | yes | 12731129 | 211 |
| cvssr3 | cactgcattggatagttgacg | atggttttgcaatggaagaag | 196/204 | (GAG)5 | Chr4 |  | 21232841 | 569 |
| cvssr4 | gtaccgcttcttcatggactc | aagctcaagtggagcgactg | 162/153 | (GGC)6 | Chr9 | yes | 22189202 | 153 |
| cvssr5 | tatggatcctcccaccaataa | gaggtgctgatgctccctac | 183/192 | (ACG)6 | Chr1 | yes | 43125263 | 192 |
| cvssr6 | ctaccgatctggggaatgag | cccatctctctctctcctcct | 141/153 | (GA)6-(GA)6 | Chr12 | yes | 22180331 | 141 |
| cvssr7 | ctacctttcgagatcggattg | atctcttctcgtcttcctccg | 101/92 | (GGA)6 | Chr8 | yes | NA | NA |
| cvssr8 | gtagactgtaggatggccacg | aagcgagcactcgttggtct | 219/201 | (CTC)5 | Chr3 | yes | NA | NA |
| cvssr9 | ctgaagccaagaaccaagaac | gccttctcttgtaccatctgc | 119/99 | (AGG)5 | Chr12 | yes | 27276103 | 99 |
| cvssr10 | aggctctcatctcgtcaaaag | aaaaagcatggtttggatcac | 101/115 | (CCT)5 | Chr11 |  | 3394458 | 1245 |
| cvssr11 | aatgatggcaactttttctcg | tcttcttgttcttgctcggac | 196/177 | (CA)6-(TC)6 | Chr8 | yes | 20529907 | 196 |
| cvssr12 | cgttgtactcttctccttggc | catgtccgagcacgtctcct | 217/208 | (CGG)5-(CGG)5 | Chr1 |  | 24629362 | 325 |
| cvssr13 | atgttcaaacaggggttggt | acacaagaacacatgcagagc | 217/209 | (CAGT)5 | Chr8 | yes | 24622714 | 209 |
| cvssr14 | gtggtatgaccggttggcct | tgaagtaggtcttgagcccg | 219/195 | (GGT)5 | Chr12 |  | 3678936 | 219 |
| cvssr15 | gggtaccagggctacttcaat | actactgctgcacagagcaaa | 214/205 | (CCG)5 | Chr8 |  | 26820729 | 1234 |
| cvssr16 | catggatttctgaagagtccg | actcccgacaccaaaatactc | 220/211 | (GCC)5 | Chr1 | yes | 27781322 | 211 |
| cvssr17 | attggattagggacagggaac | atcccattcggtagttggatt | 215/224 | (CTGC)4 | Chr1 |  | 3098 | 309 |
| cvssr18 | atgtgaatatcgcacatgctc | ttctgtatgtaatcctcccgc | 220/211 | (GCC)5 | Chr1 |  | 385844 | 802 |
| cvssr19 | atcatgaggacgcagggaac | atcacatgtcatccgctctct | 148/175 | (GCG)5 | Chr2 | yes | 22742837 | 175 |
| cvssr20 | gagggctgcagctactacgc | gaagcggaaaaatgaaaaaga | 217/209 | (CCA)6-(CG)10 | Chr2 |  | 30383068 | 209 |
| cvssr21 | gaatcattagctgttgccaaa | atcaaacatccatccatccat | 211/198 | (GATG)4-(AG)6-(GATG)4 | Chr5 | yes | 801704 | 198 |
| cvssr22 | tgttcattttgtcagggatga | tagtgacaagctgaagaccga | 150/142 | (TC)9 | Chr9 | yes | 15369354 | 146 |
| cvssr23 | Tatcttagctagctgggctgc | ccttcatacacacaccactcg | 109/79 | (GCT)6 | Chr7 |  | 29617010 | 298 |
| cvssr24 | cactagtttgcctccaagacc | cgcagagttccttcatacaca | 126/156 | (GCT)6 | Chr7 |  | 29617005 | 344 |
| cvssr25 | gagctggacctctgcctcac | gatcaggatcagctgaagagg | 173/164 | (GGC)6 | Chr3 | yes | 22977183 | 164 |
| cvssr26 | agctaggcacttcgctcctc | atagtcgtcctccatctccg | 203/194 | (CCG)5 | Chr5 |  | 29541173 | 194 |
| cvssr27 | gtacttgccggagtccatcc | tgctcgaaatctctcatgttg | 146/172 | (CCA)6 | Chr10 |  | 2844035 | 140 |
| cvssr28 | gcttgctctcctcctctcttc | ttcttgccaaagtttcccata | 204/224 | (GGTC)4 | Chr11 |  | 847527 | 812 |
| cvssr29 | aactctccgatctcacaaaaa | tcaagaaggaggaggaggag | 220/207 | (ATCG)5 | Chr9 |  | 14020399 | 205 |
| cvssr30 | cctgagatttacgacgacgac | acgtccaacctaagcccgag | 219/210 | (GCG)6 | Chr3 | yes | 8921758 | 210 |
| cvssr31 | tagctcaagcaaagggagaga | gagccccatcaagatcctac | 212/221 | (CCG)5 | Chr12 | yes | 26625841 | 221 |
| cvssr32 | tgttcttgtgctccttctcct | acaacaggtcaggggctaac | 189/162 | (CCG)5 | Chr4 | yes | 20935537 | 157 |
| cvssr33 | agcggagaaagcagtgtaatc | atagggaggatcccaacctta | 148/160 | (GCA)6 | Chr6 |  | 23467254 | 746 |
| cvssr34 | cttcggattaaaaccctagcc | cggacttcttcgacaccacc | 216/231 | (GA)6-(GC)7-(CGC)5 | Chr10 | yes | 17210268 | 314 |
| cvssr35 | ccttcgtcttggaccccatc | cccctgcagtagaagaggag | 139/119 | (GCGA)5 | Chr9 | yes | 20980533 | 119 |
| cvssr36 | agccatgcacagttatcacaa | tcccagtcctcttcagaccta | 208/220 | (AT)7 | Chr11 | yes | 27672370 | 218 |
| cvssr37 | cagcttctccaaattacgctc | gaaaggggaaaaagaaggaaa | 191/175 | (CT)7 | Chr5 | yes | 4415891 | 175 |

Chr – chromosome, V – Vandana, C – Cocodrie, amplicon – expected size (in base pairs) on the genome of rice, coordinate – physical location in rice genome of related to one flanking primer.
