# Supplementary material for: Genetic Mapping of Quantitative Trait Loci for Grain Yield under Drought in Rice under Controlled Greenhouse Conditions
Source: Front Chem. 2018 Jan 8;5:129. doi: 10.3389/fchem.2017.00129 (PMC5766644; doi:10.3389/fchem.2017.00129)
Supplement: Supplementary Table S2 — Single nucleotide polymorphism (SNP) markers placed in the genetic map of Cocodrie/Vandana F2 progenies. [file Table2.DOCX]

**Supplementary Table S2**. Single nucleotide polymorphism (SNP) markers placed in the genetic map of Cocodrie/Vandana F_2_ progenies

| SNP ID | Coordinate | Chr | Forward Primer  (Cocodrie allele) | Forward Primer  (Vandana allele) | Reverse Primer |
| --- | --- | --- | --- | --- | --- |
| S03 | 5729 | 1 | ttgttgagttgagccccaccgc | gccttgttgagttgagccccagcta | tcttatcacgcccacggcgg |
| S05 | 5795 | 1 | accatattgcaccaactagttgataacctgtacc | ccatattgcaccaactagttgataacctgtggt | ttgaatgttctttgatcctattctgaactcagtgtt |
| S23 | 15786 | 1 | ggttggtgggtgaagaaaaccacat | ggttggtgggtgaagaaaaccacac | gcaagggtcatccatcccagtgaa |
| S15 | 15574 | 1 | gcccctttagaaaatttggtgacaaagctaga | gcccctttagaaaatttggtgacaaagctagt | tttcttttaattttgatgtgaaactaaacacagtct |
| S16 | 15597 | 1 | cgcatagctggatcgaggaaaatct | gcatagctggatcgaggaaagccg | tcgtttggtactcaccgggagcttt |
| S14 | 15562 | 1 | ctcggttctaacctttatcccatcctctgt | agctcggttctaacctttatcccatcctctac | ggaccagcaagcaaaatgtgcca |
| S13 | 15558 | 1 | acacagccattaatgtgcgaggttc | acacagccattaatgtgcgagggag | cttcgtctcgccttccaaattccaa |
| S11 | 15416 | 1 | gatggaatgtttttgtcctctactattaaatgtttg | gatggaatgtttttgtcctctactattaaatgttta | ttgtggcaaattcccaacgtatccttattta |
| S25 | 15873 | 1 | aatgttcaacacatctgcggtgcag | aatgttcaacacatctgcggtaccc | ggccataagcctcggagatgaatga |
| S26 | 15885 | 1 | atggtcgacgccggagacca | atggtcgacgccggagtgcc | cagtgtcctcccgaaacagcaagc |
| S27 | 15968 | 1 | cgttctcgcgtggcgtggtag | cgttctcgcgtggcgtggtat | gtggtggtggtccgtgtccgtt |
| S39 | 17343 | 1 | gcaatgtgtcactagctagctaggtcatgttg | gtgcaatgtgtcactagctagctaggtcatatcc | cactccagcttggaactcttgccg |
| S33 | 17220 | 1 | ctgacaatgtgtgaaacaaaacgcg | ctgacaatgtgtgaaacaaaacgca | cagcagcggggccatatatacgtg |
| S38 | 17334 | 1 | attacccggtgtaacgcacgcgt | tacccggtgtaacgcacggcc | cgtcgcaggaaaattgaagggga |
| S40 | 17344 | 1 | ttcttcttcgtcgtcaccgccg | agttcttcttcgtcgtcaccgcct | tgcactgttgcacgtagctctcagc |
| S31 | 17143 | 1 | gcgtctgggtcaaaatatggctatt | gcgtctgggtcaaaatatggcttac | tgcgagagctttatttgcctttgcc |
| S36 | 17315 | 1 | acaggtgaaggaaacagatccgctt | acaggtgaaggaaacagatcctccg | ccgttgggcgggtgaaagagtt |
| S70 | 106248 | 6 | ctctacctgctccctccacctcgg | cctctacctgctccctccacctcta | agtcccggaggcgaggctagg |
| S72 | 106410 | 6 | gaaattattactccctccgtacgctgca | cgaaattattactccctccgtacgctgct | tcgttgacttctgtattttgattaacgtttgacc |
| S55 | 139200 | 9 | caagttgattgtcagttcaactttttgtcctaca | ccaagttgattgtcagttcaactttttgtcctact | acattatgatcccagtcttgaaggaatgca |
| S59 | 139754 | 9 | tggagatttgggacgacatatgaac | tggagatttgggacgacatatggtt | tacaaggcccatgcccattgttgt |
| S58 | 139752 | 9 | ggagccgttggatcttcataaacga | ggagccgttggatcttcataagggc | ttcatgcgttacagtgggaaaatga |
| S61 | 139758 | 9 | gctagcattagggaataaacccggc | gctagcattagggaataaaccccga | gcgttcaaaggcatctttcgctgtt |
| S68 | 140074 | 9 | agataaagctggtgtgcactgaaagtagtgagag | agataaagctggtgtgcactgaaagtagtgagat | cctccgcaggtaagattgaagtgcaa |
| S67 | 140067 | 9 | ttgtgtcatggattttgtcctggat | ttgtgtcatggattttgtccttgcg | tggtcaacagcatcatgcaccaaga |
| S100 | 31078 | 11 | aggctaatcaaaatgcgaggcataa | aggctaatcaaaatgcgaggcttcg | gttgctgtgagttgttggctgcgt |
| S103 | 31350 | 11 | cgagcgttcattgtccactaatgtcaca | acgagcgttcattgtccactaatgtcact | tgcagttcattagtcgatgcagtggaag |
| S102 | 31348 | 11 | gtgtgtgttctgggatgggaatgag | gtgtgtgttctgggatgggaatgaa | acaatgaacgctcgtcccgagct |
| S108 | 34876 | 11 | atggtgatctccgaccaccgcatat | gatctccgaccaccgcgcag | agtcggtcaggcaacgggca |
| S113 | 34994 | 11 | ggagggatgctccagcaggctt | gagggatgctccagcaggcga | acacaggccaagcatccaaacagg |
| S114 | 35023 | 11 | gtccacgaaacaacaacccaaatcc | gtccacgaaacaacaacccaagttg | tttgtgcgaccttagcccggtg |
| S117 | 35064 | 11 | gatcgatccgcttgtatccctaacg | gatcgatccgcttgtatccctgatt | cgtgtccgggtgggtggtgt |
| S81 | 45813 | 12 | cttgtcgtgtgggtatacacccagt | cttgtcgtgtgggtatacaccaacc | agtgagaggagttctggcgctccc |
| S88 | 46134 | 12 | ctgggagggtagaatgatcactggg | ctgggagggtagaatgatcactcgc | caaatctggtgcaggaccctgttca |
| S89 | 46160 | 12 | gcccttaaagattgcttgcagatga | gcccttaaagattgcttgcagaact | acacccactcgtgttcatccaccac |
| S94 | 46639 | 12 | gcgtcggagacggatagagaacgg | agcgtcggagacggatagagaacta | ttgagtgtggcgagaaggagcattc |
| S96 | 46771 | 12 | gcctgctgcagttttggcgtct | cctgctgcagttttggcgctg | tcataggaccttgtgtgcacggttt |

Chr – chromosome, coordinate – physical location in rice genome of related to one flanking primer.
