# Supplementary material for: Genetic Mapping of Quantitative Trait Loci for Grain Yield under Drought in Rice under Controlled Greenhouse Conditions
Source: Front Chem. 2018 Jan 8;5:129. doi: 10.3389/fchem.2017.00129 (PMC5766644; doi:10.3389/fchem.2017.00129)
Supplement: Supplementary Table S3 — Markers associated with grain yield under drought stress in F2:3 families of Cocodrie/Vandana from single marker analysis and mean grain yield (g) per plant on each genotype (A, homozygous for Vandana alleles; B, homozygous for Cocodrie alleles; H, heterozygous). [file Table3.DOCX]

**Supplementary Table S3:** Markers associated with grain yield under drought stress in F_2:3_ families of Cocodrie/Vandana from single marker analysis and mean grain yield (g) per plant on each genotype (A: homozygous for Vandana alleles; B: homozygous for Cocodrie alleles; H: heterozygous).

| **Marker** | **Chr.** | **Position (cM)** | **P value** | **LOD** | **PVE (%)** | **Add.**  **effect** | **Dom. effect** | **Gene action** | **Mean-A** | **Mean-H** | **Mean-B** |
| --- | --- | --- | --- | --- | --- | --- | --- | --- | --- | --- | --- |
| RD0107 | 1 | 36.5 | 0.0005 | 3.36 | 3.89 | -0.03 | -0.48 | OD | 2.84 | 2.39 | 2.90 |
| RM17710 | 5 | 0 | 0.0005 | 3.16 | 3.53 | 0.34 | -0.080 | Add. | 3.01 | 2.60 | 2.33 |
| cvssr21 | 5 | 4.2 | 0.001 | 2.80 | 3.14 | 0.34 | -0.07 | Add. | 3.007 | 2.597 | 2.321 |
| RM17779 | 5 | 4.4 | 0.0005 | 3.06 | 3.43 | 0.35 | -0.04 | Add. | 3.007 | 2.613 | 2.302 |
| RD0806_4 | 8 | 61.1 | 0.005 | 2.93 | 3.42 | 0.29 | -.06 | Add. | 2.907 | 2.561 | 2.318 |
| RM23017 | 8 | 59.5 | 0.01 | 2.63 | 2.96 | 0.27 | -0.09 | Add. | 2.898 | 2.536 | 2.360 |

Chr. – chromosome, cM – centi Morgan, Mbp – mega base pair, LOD – logarithm of odds, PVE – phenotypic variance explained, add. – additive, dom. – dominance, OD - overdominance
