# Supplementary figures and images for: Genetic Mapping of Quantitative Trait Loci for Grain Yield under Drought in Rice under Controlled Greenhouse Conditions
Source: Front Chem. 2018 Jan 8;5:129. doi: 10.3389/fchem.2017.00129 (PMC5766644; doi:10.3389/fchem.2017.00129)

## Slide 1
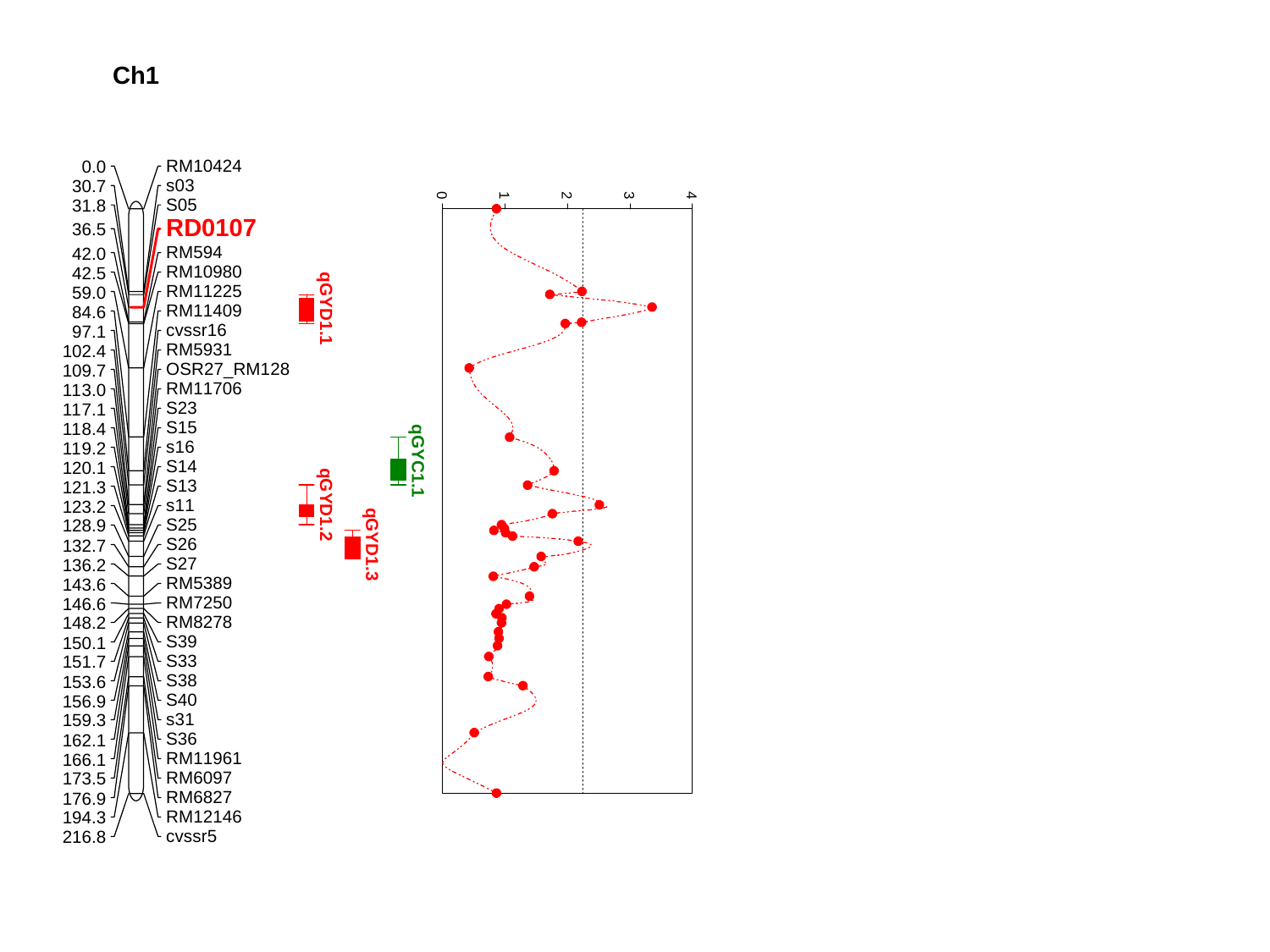

## Slide 2
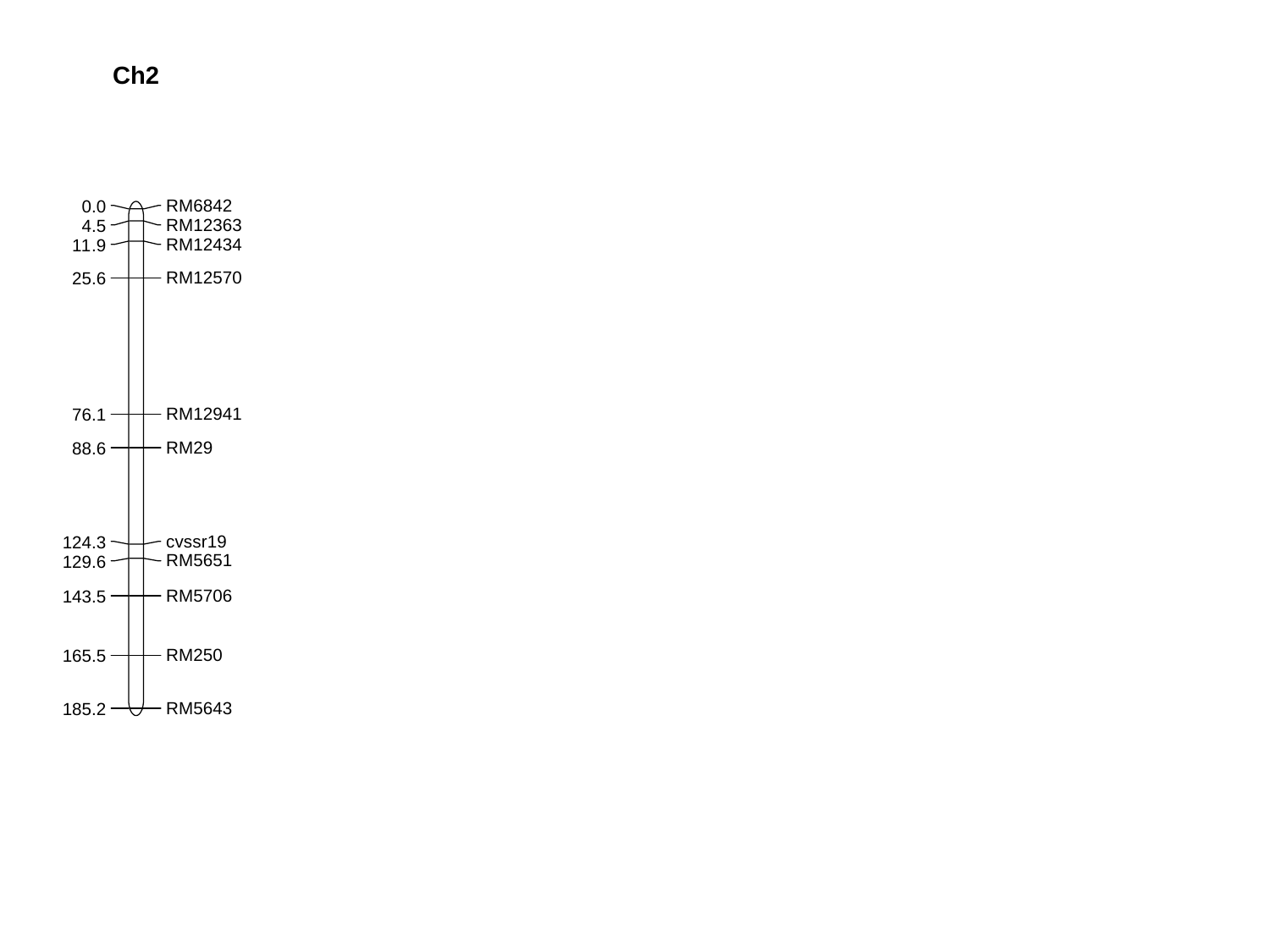

## Slide 3
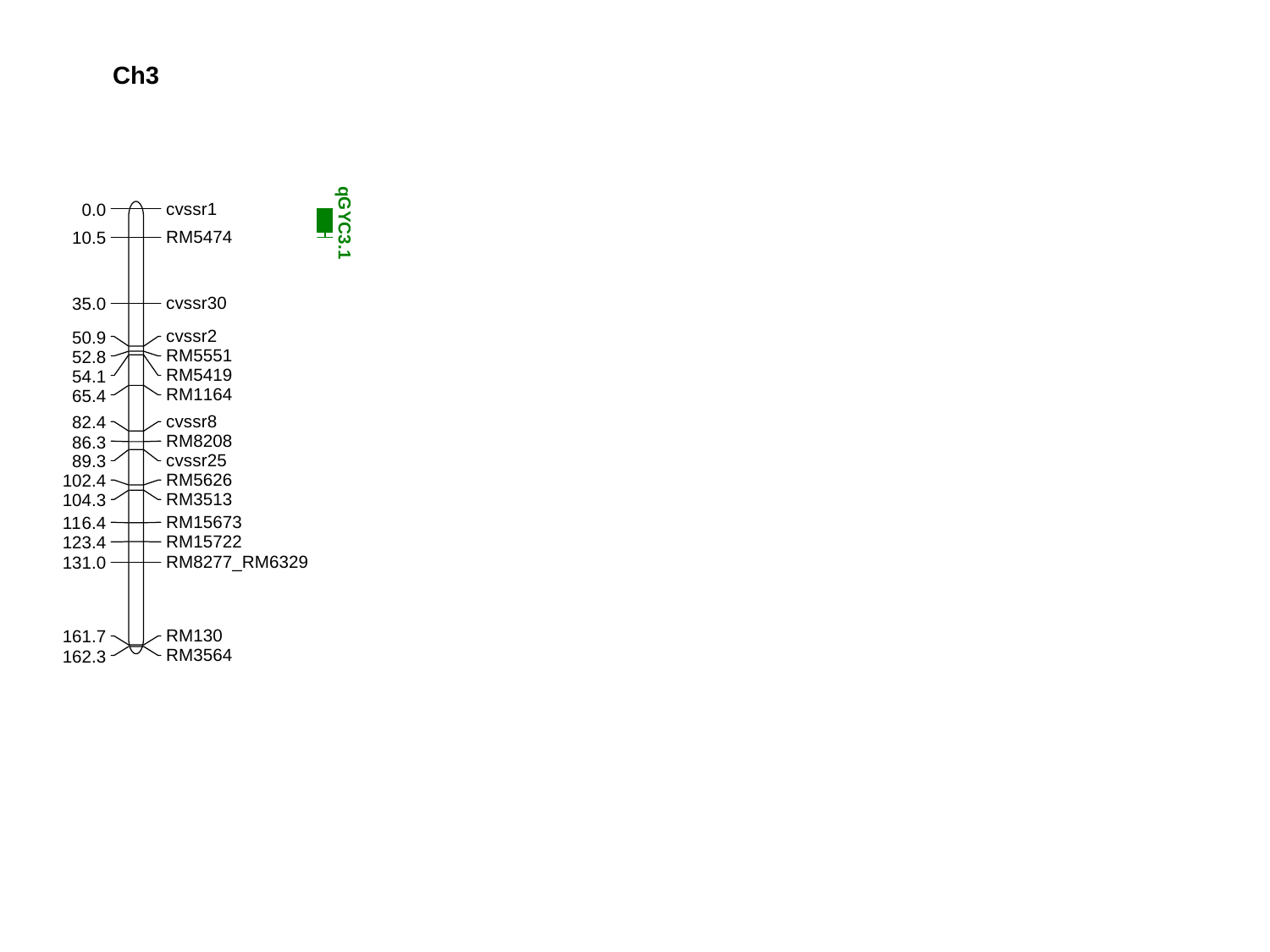

## Slide 4
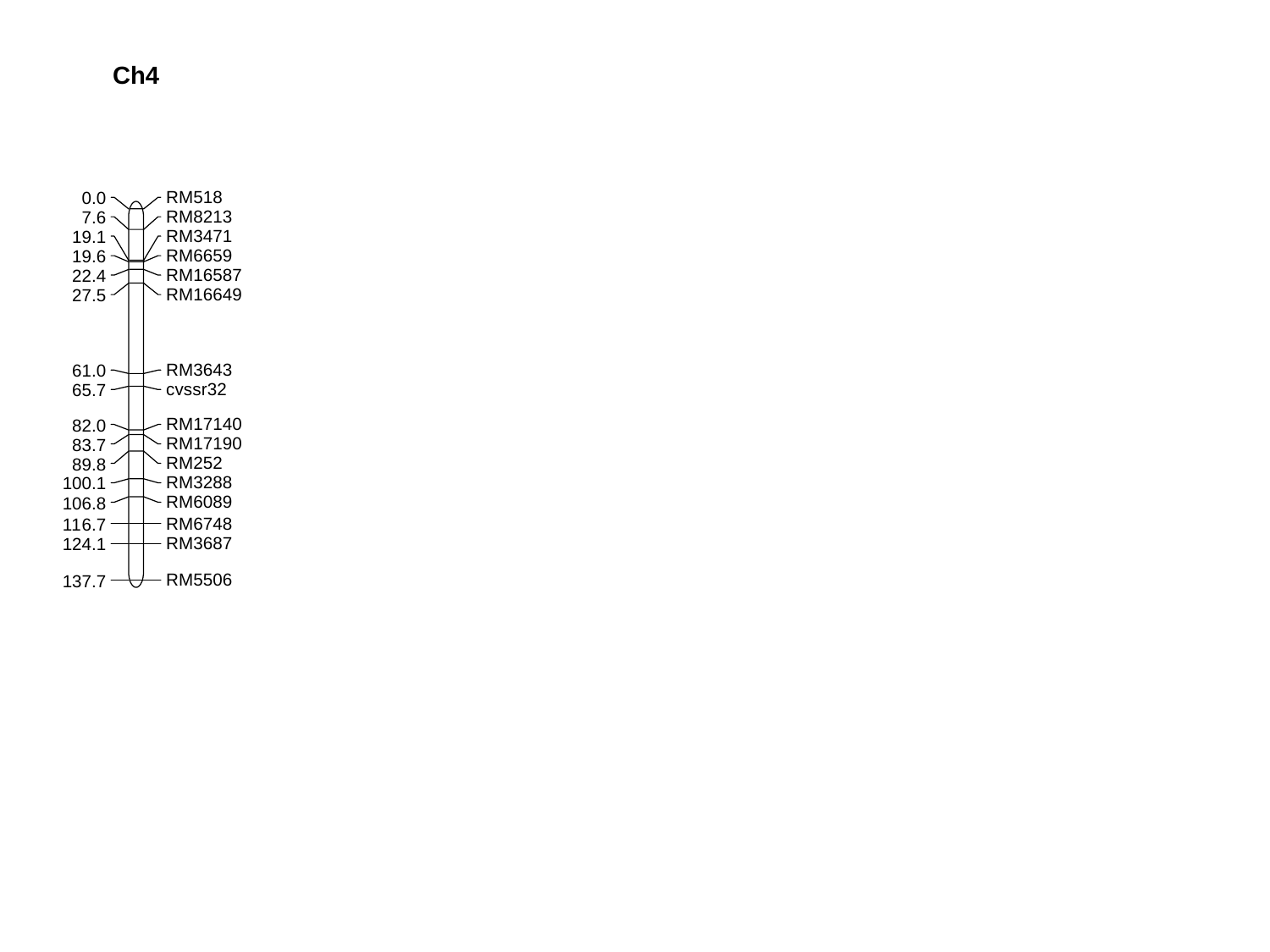

## Slide 5
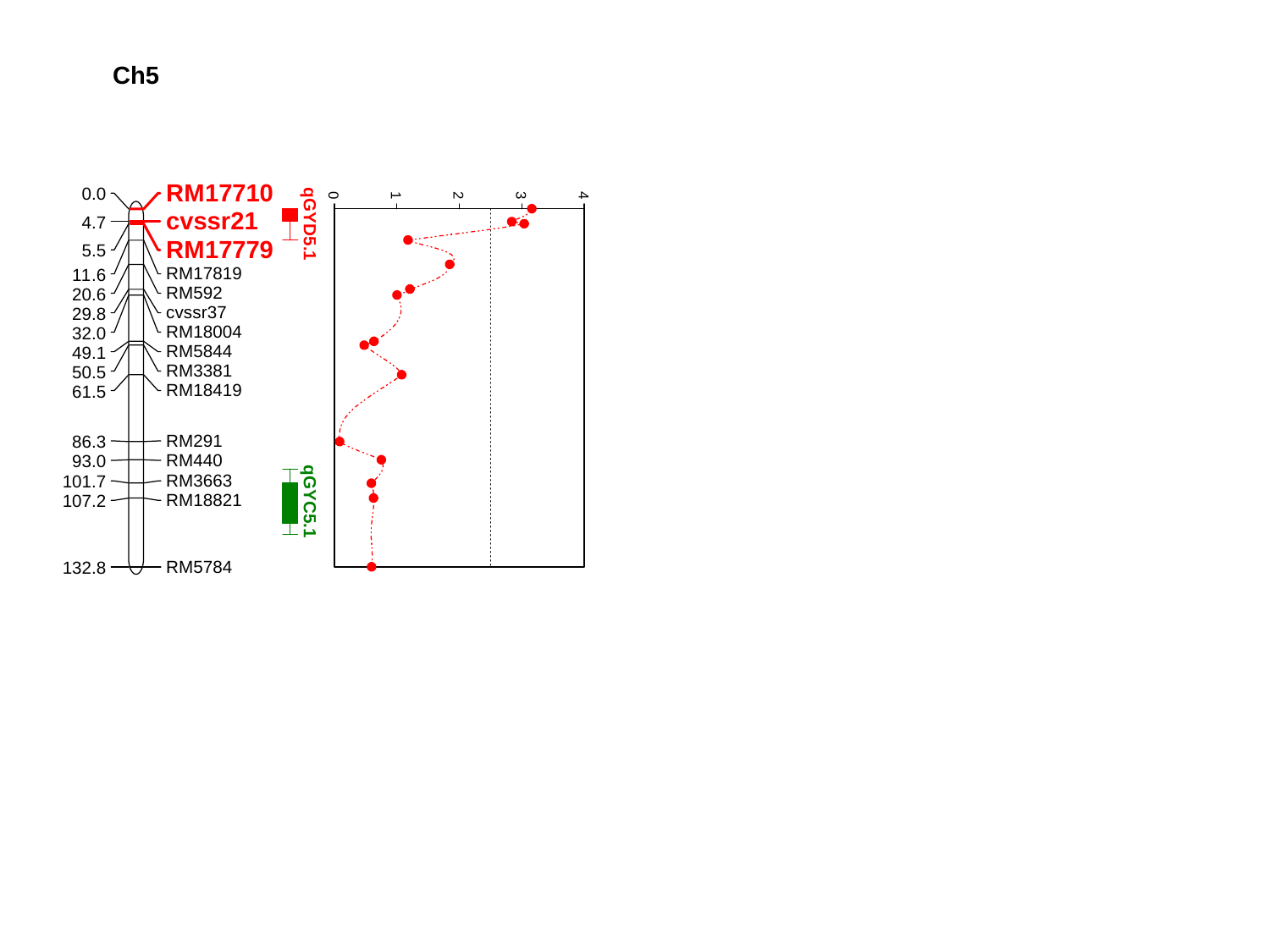

## Slide 6
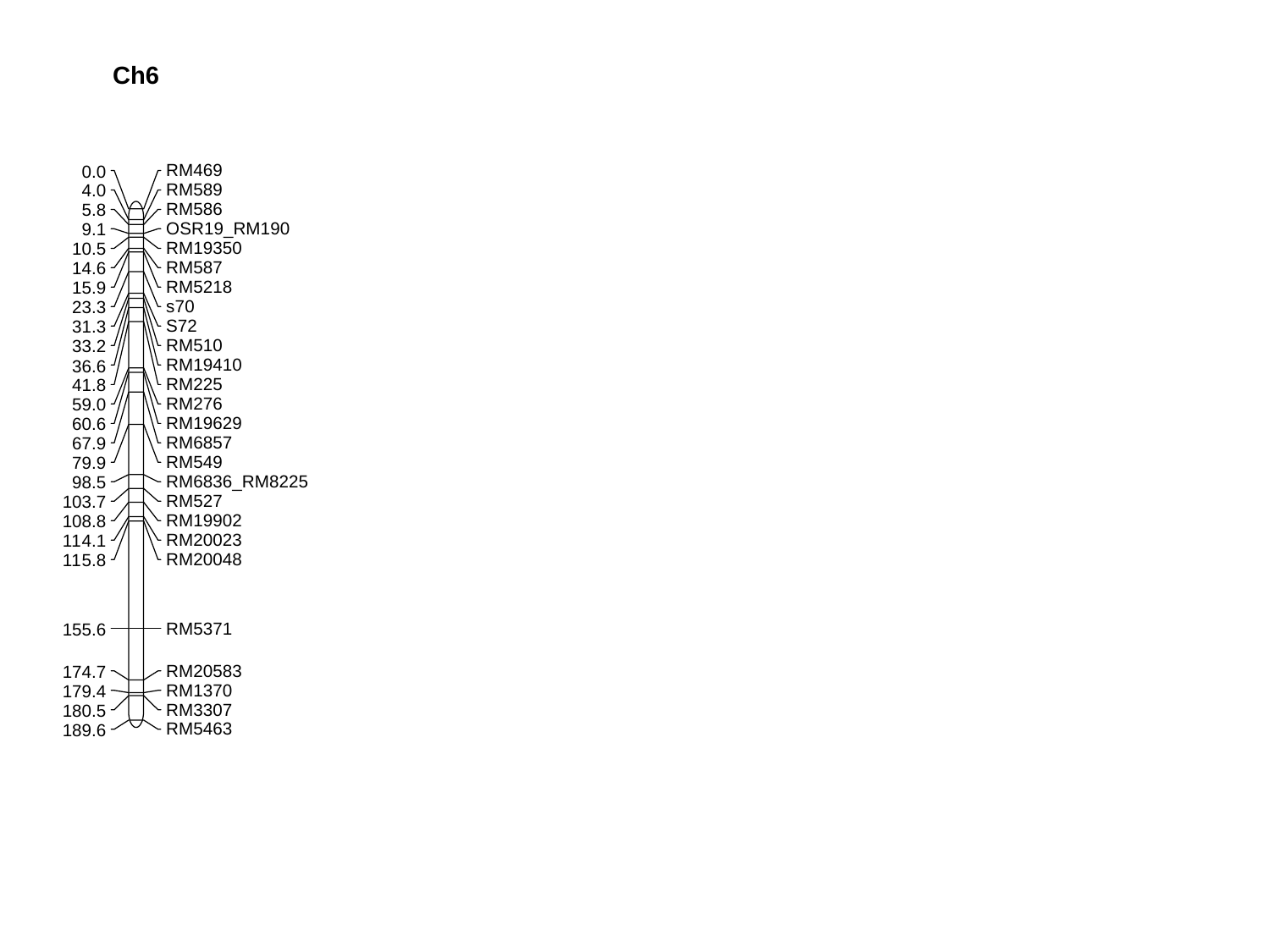

## Slide 7
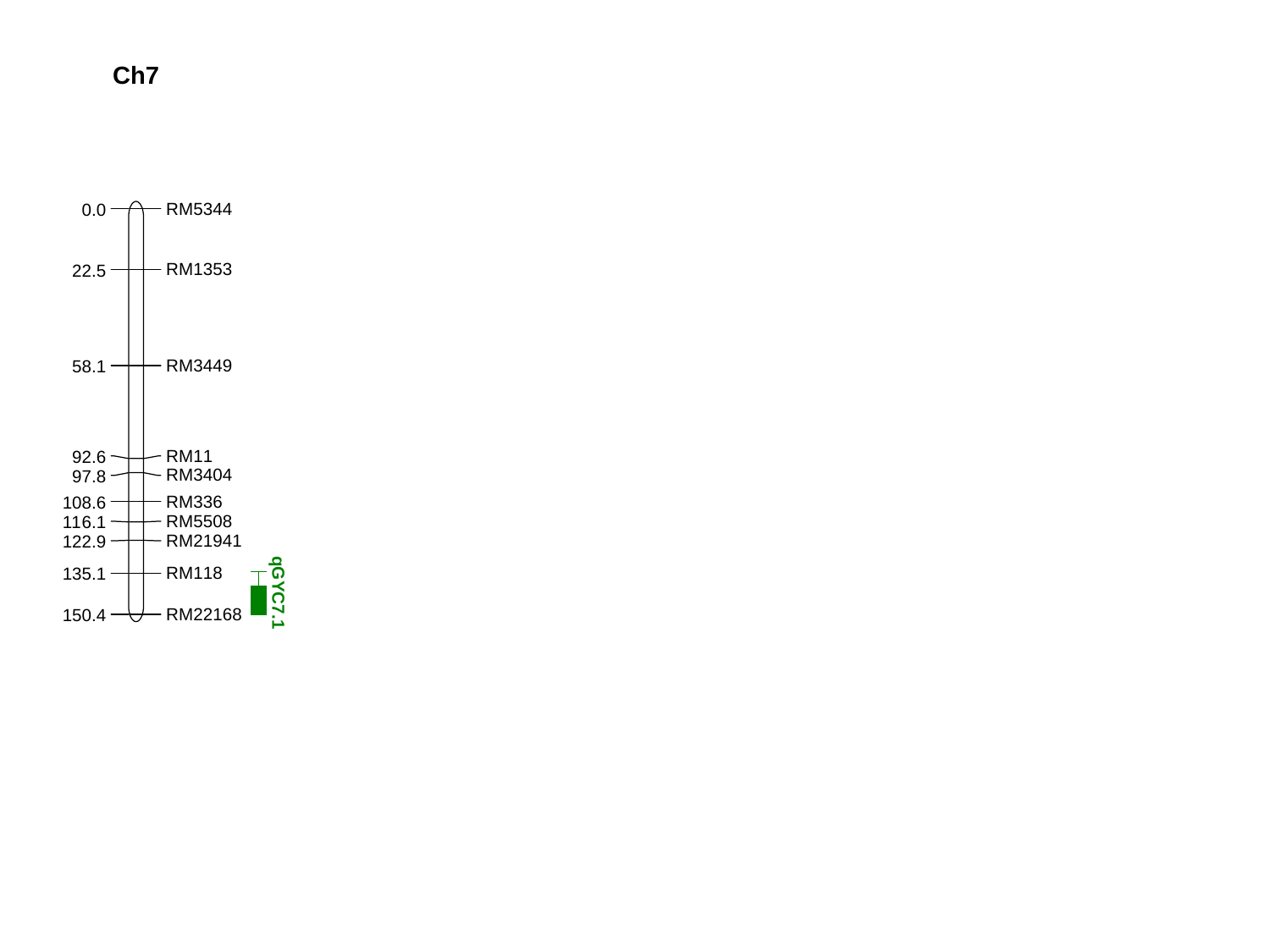

## Slide 8
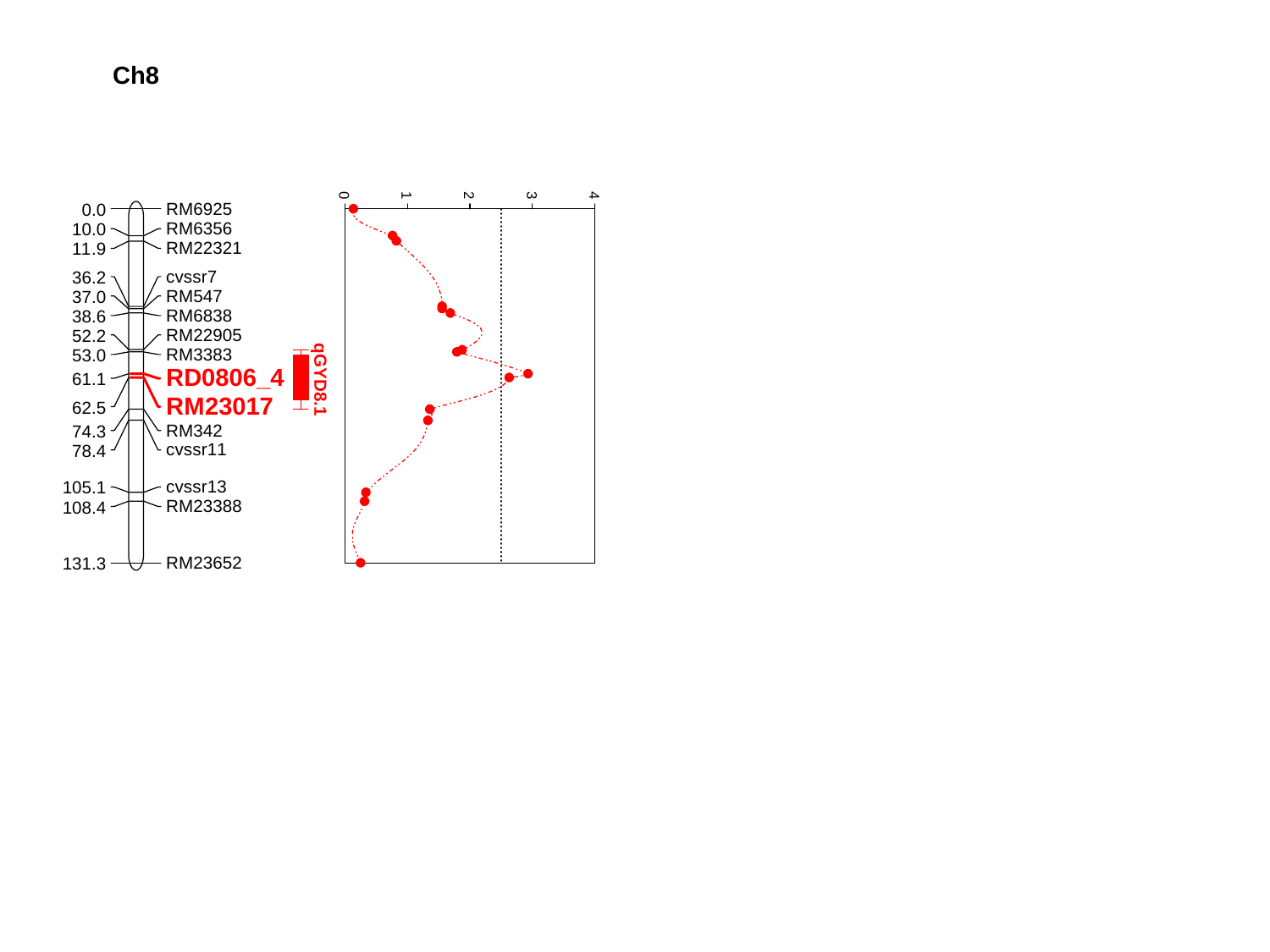

## Slide 9
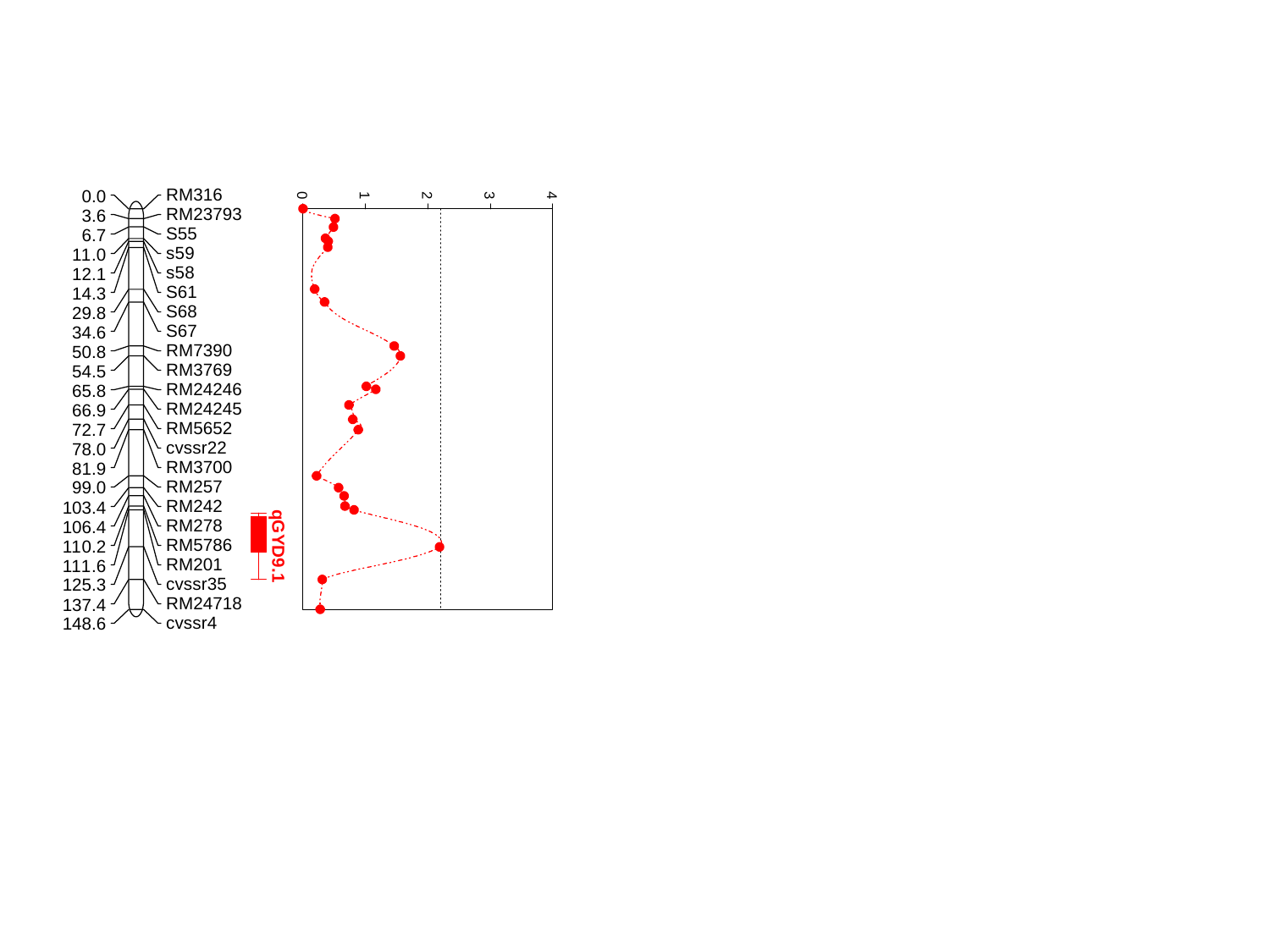

## Slide 10
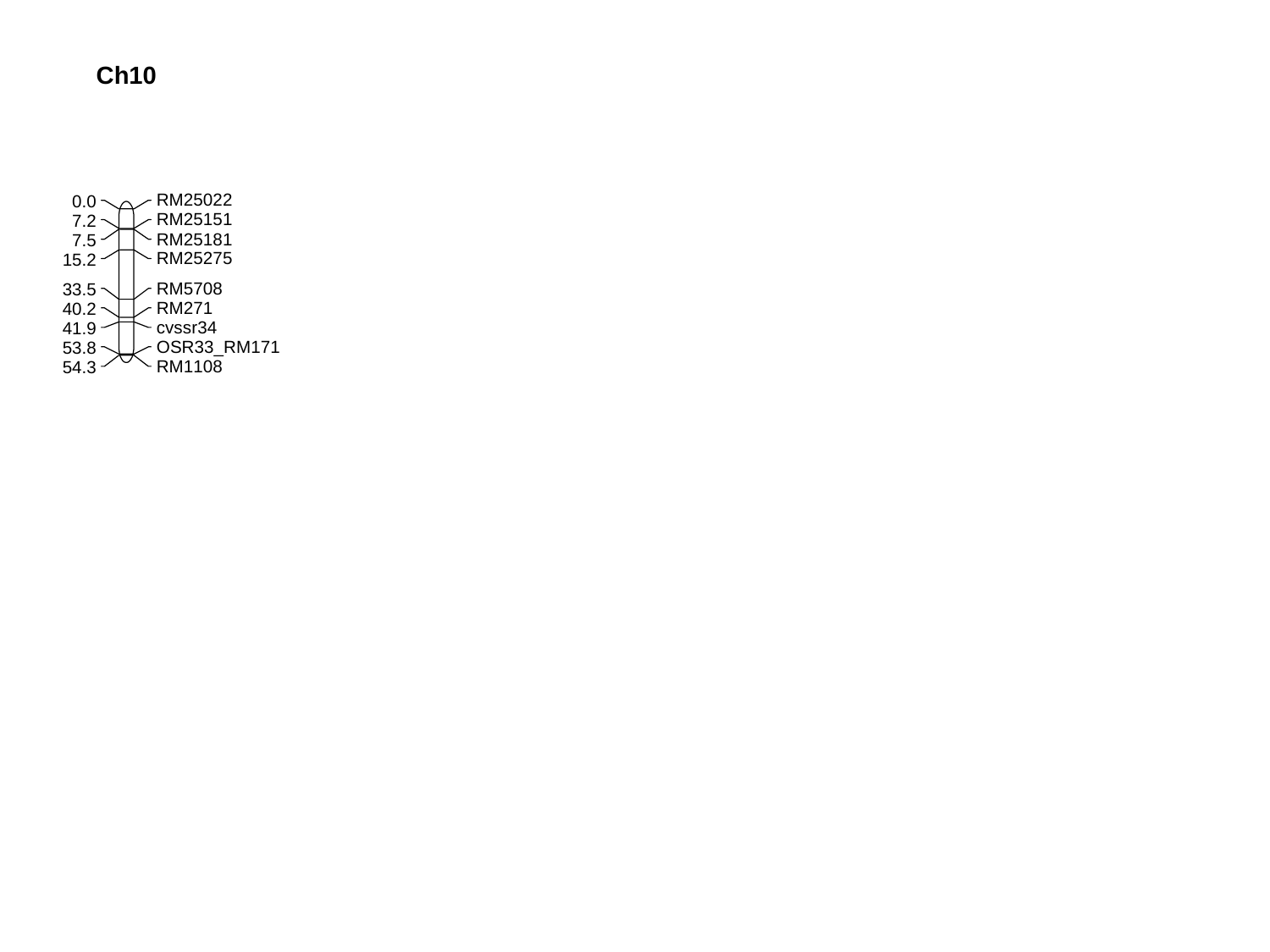

## Slide 11
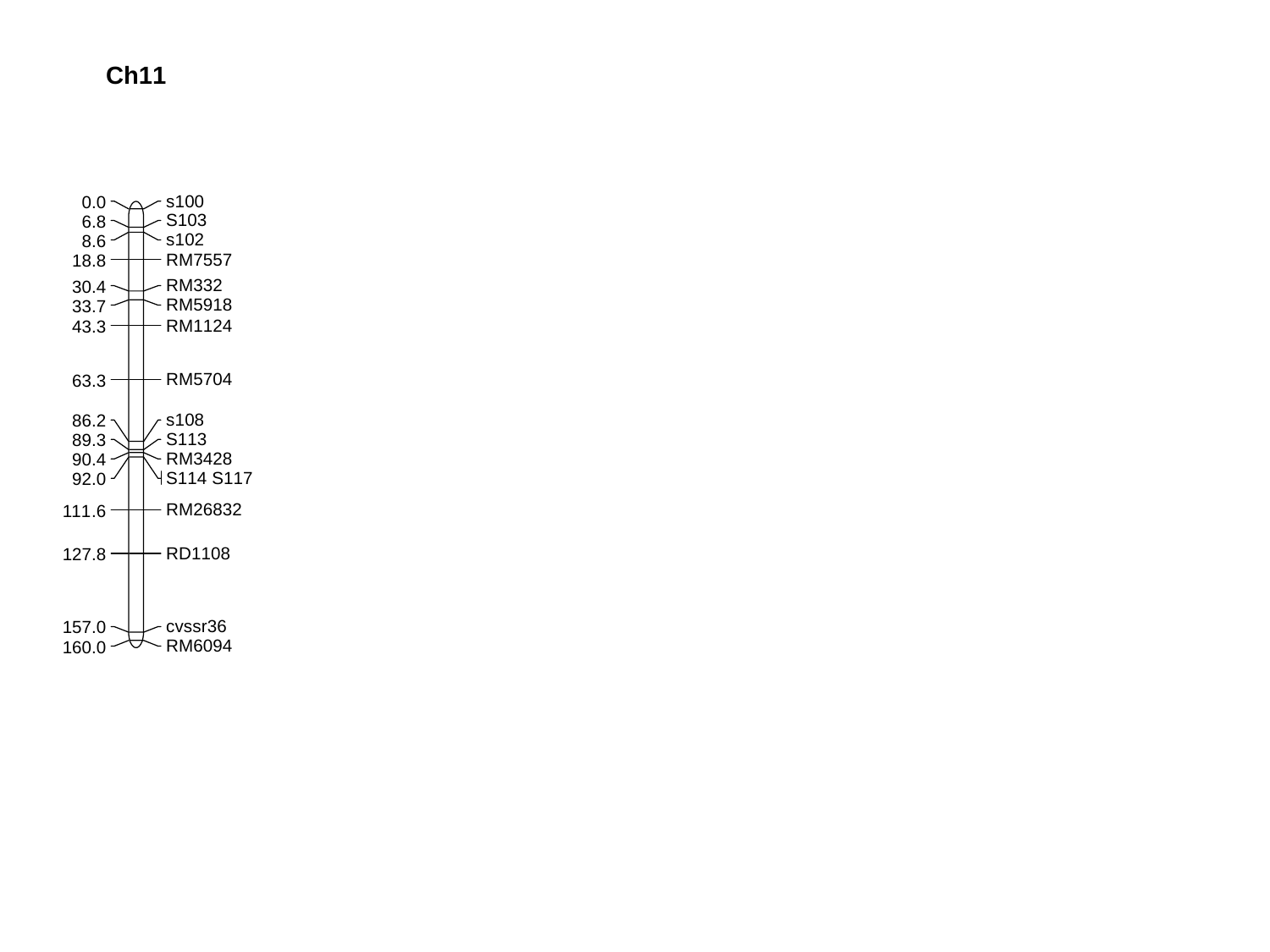

## Slide 12
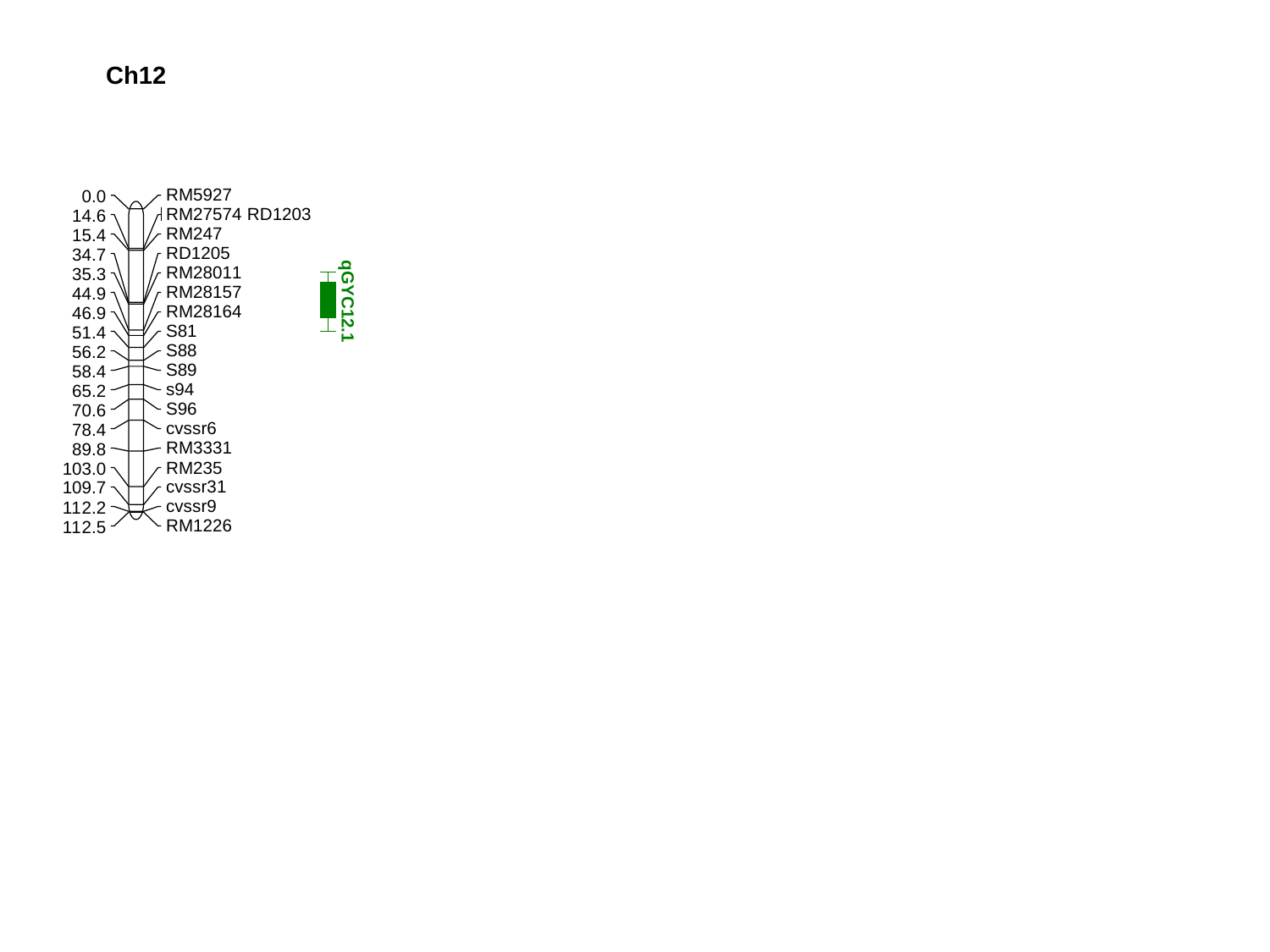

Supplement: Supplementary Figure S1 — Linkage map of the F2 individuals showing distribution of the markers on 12 chromosomes. QTLs for grain yield under greenhouse drought are shown in red rectangles and QTLs for grain yield under non-stressed control are in green rectangles. Markers identified through single marker analysis and within the QTL interval are depicted in bold red fonts. [file Presentation1.PPTX]
